# What are the impacts of activities undertaken in UNESCO biosphere reserves on socio-economic wellbeing in Southeast Asia? A systematic review

**DOI:** 10.1186/s13750-023-00322-1

**Published:** 2023-12-14

**Authors:** Nguyen Phuong Thao, Jacqualyn Eales, Duong Minh Lam, Vu Thuc Hien, Ruth Garside

**Affiliations:** 1 Hanoi National University of Education, 136 Xuan Thuy Street, Cau Giay District, Hanoi, Vietnam; 2 European Centre for Environment and Human Health, Peter Lanyon Building, University of Exeter Penryn Campus, Penryn, TR10 9FE Cornwall UK; 3 Vietnam Man and Biosphere Program National Committee, Hanoi, Vietnam

**Keywords:** Conservation, Livelihood, Sustainable development, Biodiversity, Economic development

## Abstract

**Background:**

UNESCO biosphere reserves (BRs) have historically aimed to play a crucial role in contributing to sustainable development by bringing about win–win outcomes for both biodiversity and socio-economic development. However, recent studies show the need for a more thorough understanding of how conservation activities impact on and are affected by socio-economic development.

**Method:**

We built this systematic review on a systematic map by Eales et al. [14] adding studies from further academic database and grey literature searches specifically designed for this systematic review. Because studies were not sufficiently homogeneous in their outcomes to warrant a valid meta-analysis, we used narrative synthesis to explore the studies’ findings.

**Results:**

We assessed 10,053 titles and abstracts from database searches and Google Scholar. 343 articles were screened at full text and 16 studies were included in our review. Of the 16 studies, 3 were assessed as having overall high validity, 8 having moderate validity and 3 having low validity of evidence. 2 studies did not provide sufficient information for validity categorisation (unclear validity). Effects on economic living standards, reported in 11 studies, were in both desired and undesired directions, though most high validity studies reported no significant difference, and most others did not test for significance. Most studies reported that BR interventions were associated with positive impacts on material living standards. In general, studies reported good relations between local people and local enforcement/government following interventions in BRs. BR interventions may both reduce or cause social conflict, though the higher validity studies showed results in the desired direction. In one study, there was a positive impact on population family planning outcomes, when a reproductive health intervention was implemented with conservation efforts. There was no clear impact in either direction regarding education. Across two studies the overall message is positive for the subjective wellbeing of local people.

**Conclusions:**

With 727 BRs worldwide, the BR model has been accepted and developed as an approach to facilitate the implementation of the UN's SDGs. However, our work shows that interventions implemented in UNESCO BRs can bring about impacts in quite diverse ways: positive, negative, unchanged, and may often present both positive and negative impacts in the same situation. This reconfirms that the expected win–win outcomes of UNESCO BR model in terms of biodiversity and socio-economic development should be more carefully considered. We suggest some main points for consideration, particularly when developing management mechanisms for UNESCO biosphere reserves and/or managing activities in biosphere reserves. We also highlight the need for further research to explore the socio-economic impacts of the UNESCO biosphere reserves in Southeast Asia, especially on the domains of freedom of choice and action, security and safety, and culture and spirituality. Moreover, it is vital to have research projects that measure long-term impacts of biosphere reserves, which have been lacking in previous work. Finally, the potential impact of external factors should be considered in programme and monitoring design.

**Supplementary Information:**

The online version contains supplementary material available at 10.1186/s13750-023-00322-1.

## Background

Southeast Asia (SEA) is renowned for its high biodiversity [31] and vast amount of carbon stored in peatlands [1]. However, tropical forests in this region have experienced a high rate of forest loss, especially beginning during the 1990s [16], driven mainly by industrial agriculture [31]. The current rapid rate of deforestation in the region has resulted in serious global consequences [31]. The heart of SEA is a global biodiversity hotspot where most tropical marine groups have their greatest density of species [8]. However, the vast majority of SEA’s reefs are also at risk [5] due to the fact that "burgeoning human populations are over-utilizing the resources in many areas, while wholesale destruction of the forests on land, together with rapid urbanization, is leading to massive loads of sediments and pollution" [34], p. 259]. Conflicts over marine ecosystem resources are growing in SEA, particularly as many people in the region live and depend on the coast [23] for their livelihoods. In this context, UNESCO biosphere reserves, which ban or restrict destructive activities in core zones, and promote solutions reconciling the conservation of biodiversity in a culturally and economically sensitive manner, are expected to play a crucial role in contributing to sustainable development regionally and globally.

The concept of Biosphere Reserves was introduced in 1975 [19] by UNESCO in response to the need for conservation of biodiversity whilst ensuring its sustainable use. Biosphere reserves comprise terrestrial, marine and coastal ecosystems for the purpose of preserving genetic diversity^1^ in representative ecosystems by protecting wild animals, the traditional lifestyle of inhabitants and domesticated plant/animal genetic resources [19]. Currently, SEA is home to 35 UNESCO biosphere reserves.^2^ Biosphere reserves typically comprise three zones. In the core zone, all exploitation of natural resources is banned (including timber and non-timber forest products and wildlife), whilst only activities of forest management and protection, wildlife conservation, research, and education are permitted. In the buffer zone and transition zones, sustainable socio-economic development activities, research, education, and entertainment are permitted, and the development of industrial zones, mining, large construction projects is limited. It is worth noting that the management mechanisms can be varied and differ between BRs even in the same country. For example, in Vietnam, local people are not permitted to live in the core zones of BRs of Kien Giang, and Ca Mau, however, in Cu Lao Cham-Hoi An and Dong Nai BRs, local people still live in the core zones because they settled there before they were designated. Local people living in the core zone must comply with many regulations that aim to protect biodiversity. This type of arrangement/management aims to balance the needs of local people with core zone protection.

This model of natural conservation has been expected to bring about win–win outcomes for both biodiversity and socio-economic development [3, 35]. However, some research shows a lack of thorough understanding of how conservation can impact on, and be impacted by socio-economic systems [6, 7, 40]. According to Woodhouse et al. [40], conservation interventions can have positive impacts on the local economy through generating jobs and alternative livelihoods, yet could negatively impact other social aspects of the communities, such as social relationships and autonomy. Some local communities can be particularly vulnerable, for example, the Orang Asli within the Tasik Chini UNESCO BR in Malaysia. This community faced socio-economic challenges such as low-income traps, disparate livelihood alternatives, widespread symptoms of alcoholism/substance abuse and safety and cultural integrity issues of residential areas involving tourism development, at the time of gazettement of the BR in 2009 [20].

In addition, interventions with the joint goals of conservation and human development have faced sizable challenges due to the conflicting interests induced by rapid social and environmental challenges, such as climate change. This, therefore, raises the need for a thorough understanding of the relationship between natural conservation and socio-economic development. The topic has gained increasing attention in past decades, reflected in a number of secondary research articles published recently. McKinnon et al. [21] published a systematic map with 1043 articles to document the impacts of natural conservation interventions on different domains of human wellbeing in developing countries. Ban et al. [4] reviewed 118 articles to analyse wellbeing outcomes related to marine protected areas on a global scale with a focus on both positive and negative impacts on people. In a systematic map, Eales et al. [14] found 287 research articles on the impact of marine management and conservation interventions on human wellbeing of coastal communities in SEA. To be recognized as a BR by UNESCO, countries must submit a proposal, which shows their natural, biological, cultural, economic, political and development plans, as well as a commitment to ensure that the plan is implemented. From conception, BRs aim to follow the UN sustainable development goals as well as those of each country.

The management strategies of the BRs are diverse and vary between localities and countries. However, across all BRs, a resounding feature is that the management of BRs is typically challenging. Managers and policies often necessarily attempting to meet the needs of the environment and people, connecting often disparate stakeholders with conflicting goals, which can raise the stakes and has the potential to raise conflict without careful stewardship of implementation. The benefits of the BR being a relatively open model of management does however allow a somewhat flexible a management mechanism which can adapt to specific contexts.

There are now 727 BRs worldwide, 35 in Southeast Asia. In 2021, following the development of UNESCO BR designation requirements, some BRs no longer qualified and were withdrawn from the UNESCO BR list.

This systematic review analyses the impact of activities conducted in BRs on socio-economic development. We do not aim to understand the efficacy of UNESCO BRs themselves, rather, the impact of activities conducted within them; activities which fall under the goals of BRs. This collation of evidence serves as a basis for UNESCO to improve policies for BRs in Southeast Asia as well as worldwide. We focus on SEA both due to its high levels of marine biodiversity, and as UNESCO BRs were focal case studies in the GCRF-UKRI *Blue Communities* project (2018–2021) through which this systematic review was funded.

### Stakeholder engagement

This review was conducted with the engagement of the Vietnam Man and Biosphere Program (MAB Vietnam) National Committee and UNESCO Regional Science Bureau for Asia and the Pacific, who were involved in question setting. UNESCO representatives from the regional office were asked what the evidence need was in the region. A senior programme specialist identified the need to understand to what extent interventions undertaken in UNESCO biosphere reserves in SEA impact the socio-economic development in these areas. This is recognised as having vital implications for natural conservation implementation in the future. The stakeholders suggested sources of grey literature and provided annual reports by UNESCO biosphere reserves in SEA and the reports by the Southeast Asian Biosphere Reserves Network (SeaBRnet). A scoping meeting was arranged between the review team and MAB Vietnam to discuss the potential factors affecting the success or failure of a UNESCO biosphere reserve, which informed the development of the protocol to this review [25].

## Objective of the review

This review examines the question:

“What are the impacts of activities undertaken in UNESCO biosphere reserves in Southeast Asia on socio-economic wellbeing?”.

This question includes the following key “PICO” components:

- Population: human populations in UNESCO biosphere reserves in Southeast Asia
- Intervention: activities undertaken in UNESCO biosphere reserves*
- Comparator: Where present, an eligible comparator is the same site before activities undertaken, or a site without activities (we note whether the site was designated as a UNESCO biosphere reserve at the time of the comparator), or a site with activities but outside of a UNESCO biosphere reserve. We also consider studies with no comparator.
- Outcomes: any measures of socio-economic status.

*The activities undertaken must align with the stated functions of UNESCO biosphere reserves, having the aim of one or more of the following^3^:

- Conservation of biodiversity and cultural diversity
- Economic development that is socio-culturally and environmentally sustainable
- Logistic support, underpinning development through research, monitoring, education, and training.

## Methods

### Deviations from the protocol

Several deviations from the published protocol are detailed below, along with reasoning for the deviations.

The protocol stated that we would have 100 records from each grey literature searching platform. We expanded this to 200 (50 from each of the 4 search strings), because this represented a manageable number and because it increased the probability of retrieving relevant records. We added one thesis repository site to be searched (Erasmus) to those listed in the protocol, because we became aware of it only after protocol publication. Recognising that different activities are undertaken in the three zones of a biosphere reserve, we clarified the inclusion criteria, from those set out in the protocol, stating that we include studies which investigate one or more levels of management within UNESCO reserves, e.g., core zone, buffer zone or transition zone. We added “Moderate” into the overall study validity classification categories because it enabled us to better differentiate between study validity, having four categories, rather than three, as originally proposed in the protocol.

This study is conducted as part of the GCRF UKRI-funded *Blue Communities* programme (2018–2022), aiming at building capacity for sustainable marine ecosystems for the benefit of the health, wellbeing, food security and livelihoods of coastal communities in SEA. As part of the programme, researchers undertook a systematic map to examine the impact of marine management and conservation interventions on human wellbeing in SEA [14]. This evidence scope of our systematic review differs from the map, having a narrower population/intervention (UNESCO BRs with activities aligned with programme aims), and a wider geographical scope, including both terrestrial and marine areas. We followed a protocol which was pre-published, open access in Zenodo [25] and followed the ROSES reporting criteria set out for the conduct of CEE Systematic Reviews [18].

### Search for articles

#### Overall search strategy

We considered both published and grey literature in English in this review. We conducted our searches in the following sources: bibliographic databases, web-based search engines and grey literature. We also used the database from the systematic map in Eales et al. [14] to identify relevant literature from the extensive and comprehensive searching undertaken for that work. Of the articles in the database, 160 included quantitative data, and were eligible for screening for our review. All searches were undertaken in December 2020/January 2021, with exact dates of searches provided in Additional file 1. We did not update the searches, because they were undertaken less than 24 months ago.

#### Search terms and strings

We based our search string on the population terms only, and not intervention or outcome terms, because scoping revealed a manageable number for screening using this approach. Additionally, if intervention or outcome terms were added into the search, there is a danger of potentially missing articles if study authors used outcome terms which were not included in our search term set. The rationale for focusing on names of UNESCO biosphere reserves, and the term “biosphere reserve” is that any intervention undertaken should have been done with the understanding that the site was a UNESCO biosphere reserve, and that the intervention was aiming to meet the UNESCO goals. If an article reported research undertaken in a UNESCO biosphere reserve, but did not mention the search terms below, the intervention was highly unlikely to be under the management of UNESCO or to align with the functions of biosphere reserves.

We note that there are different spellings for some UNESCO biosphere reserve names. To include these different spellings, we adapted the search string for each database, according to the wildcard capabilities of each. For example, in Web of Science, we used “Berbak$Sembilang”, which retrieves “Berbak Sembilang” and “Berbak-Sembilang”. “Tonle Sap” may also appear as “Tonlé Sap”; “Inlay Lake” may appear as “Inle Lake” and “Hauy Tak Teak” as “Haui Tak Teak” or “Huai Tak Teak”.

The search string below is formatted for Web of Science, as an example.

"Tonle Sap" OR "Tonlé Sap" OR “Cibodas” OR “Komodo” OR “Lore Lindu” OR “Tanjung Puting” OR “Gunung Leuser” OR “Siberut” OR “Giam Siak Kecil-Bukit Batu” OR “Wakatobi” OR “Bromo Tengger Semeru*” OR “Taka Bonerate-Kepulauan Selayar” OR “Belambangan” OR “Berbak-Sembilang” OR “Betung Kerihun Danau Sentarum Kapuas Hulu” OR “Rinjani Lombok” OR “Tasik Chini” OR “Crocker Range” OR “Inlay Lake” OR “Inle Lake” OR “Indawgyi” OR “Puerto Galera” OR “Palawan” OR “Albay” OR “Sakaerat” OR “Hauy Tak Teak” OR “Haui Tak Teak” OR “Huai Tak Teak” OR “Mae Sa-Kog Ma” OR “Ranong” OR “Can Gio Mangrove” OR “Dong Nai” OR “Cat Ba” OR “Red River Delta” OR “Kien Giang” OR “Western Nghe An” OR “Mui Ca Mau” OR “Cu Lao Cham*” OR “Langbiang” OR “Boeng Chhmar” OR “Prek Toal” OR “Puerto Princesa Subterranean River” OR “Tubbataha Reefs” OR “Kaper Estuary” OR “Laemson Marine National Park” OR “Kraburi Estuary” OR “biosphere reserve*”

#### Bibliographic database searches

We used four bibliographic databases: Medline, Web of Science Core Collection, SCOPUS and Environment Complete, with University of Exeter Institutional subscription. We did not include Global Health (Ovid) because it focuses on health topics. Searches were undertaken for “topic words” or “title, abstract and keywords” rather than “full text”, to limit the number of irrelevant retrieved hits. We did not impose any date cut-offs, and searches were limited by language to English only for bibliographic databases. The language limitation is unlikely to reduce sensitivity, because we expect most peer-reviewed research articles on the topic to be published in English. The dates, exact search strings and limits of the bibliographic database searches are given in Additional file 1.

#### Supplementary searches: web-based search engines

We used Google Advanced (www.google.com) and Google Scholar Advanced (www.scholar.google.com), using the search strategy below, and searched in the title of the page. In testing of search engines, we found that using “OR” between “biosphere reserve” and the names of reserves returned many irrelevant results. Using “AND” made the results more precise, so we used this Boolean operator. Due to the character limit in the functionality of Google searches, we had to undertake four separate searches for each Google platform, rather than the original single one planned, splitting the 35 UNESCO biosphere reserves into the four searches. We downloaded 200 search records from each of the platforms.

name of one of 35 UNESCO biosphere reserves in SEA

AND

“biosphere reserve*”.

We did not add any other restrictions. For Google Scholar results, we used the software “Publish or Perish” https://harzing.com/resources/publish-or-perish to download RIS files for the search results. Full details of the Google Advanced and Google Scholar Advanced search strings and dates of searches are given in Additional file 2.

#### Supplementary searches: organisational websites and theses databases

The following specialist websites of organisations were included to search for relevant grey literature:

- https://unesdoc.unesco.org/ark:
- https://jfit-for-science.asia/
- http://mabvietnam.net/

We searched 12 repository sites for relevant evidence, particularly theses and reports. The search string from the database searches was adapted to reflect the search functionality of each repository.

List of repository sites searched:

- Cybertesis
- DART-Europe
- DiVA
- Ethos
- NARCIS
- National ETD
- National Library of Australia Trove Service
- NDLTD
- Proquest Dissertations and Theses Global
- Repositorio Cientifico de Acesso Aberto de Portugal
- Theses Canada
- Erasmus Thesis Repository

We did not add any date or language restrictions. For all website and catalogue searches we recorded the URL, the strategy or search terms used, the date the search was undertaken, the results, and the name of the reviewer undertaking the search. This information is collated in Additional file 3.

#### Other methods of obtaining evidence

The stakeholders from MAB Vietnam National Committee and UNESCO provided periodic reports from UNESCO biosphere reserves. These reports had potentially useful sources of information, for example, listing research projects undertaken in the reserve, which may help to identify gaps between funded research and published research. We also undertook forward and backward citation chasing for all included studies, details can be found in Additional file 3.

#### Estimating the search comprehensiveness and managing the results

To check the comprehensiveness of the bibliographic database search, we tested the search using a benchmark list of articles pre-identified as relevant to our topic using an initial scoping search, to make sure that they are retrieved by the search, listed in Additional file 1. Four of the five articles were retrieved by the initial search strategy in Web of Science Core Collections. The one article that was not retrieved by the search strategy was because the article referred to a named site (Tubbataha reefs) within the UNESCO biosphere reserve (Palawan), rather than the reserve itself. With this knowledge we retrieved a list of multi-internationally designated sites within UNESCO biosphere reserves from our stakeholders and modified our search strategy to include these, detailed in Additional file 1.

We used the software Endnote (www.endnote.com) to collate and de-duplicate the search results from each of the four databases (and from Google Scholar) to form a library of 10,214 records. We then imported the resulting library into Rayyan (www.rayyan.ai) which enabled us to find a further 161 further duplicates, leaving 10,053 deduplicated articles.

### Article screening and study eligibility criteria

#### Screening process

The screening process was conducted in two steps by one of five independent reviewers (NPT, JE, DML, HV and DD): (1) title and abstract and (2) full text of articles.

First, the title and abstract of each article was screened based on the study inclusion criteria (Box 1). 13% (1258) of the articles were dual screened (i.e., screened by two reviewers independently) at the title and abstract screening stage. The articles seeming to meet the inclusion criteria were obtained at full text and further screened against the criteria to establish the final set of articles for reviewing. 17% (58) of the articles were dual screened at the full text screening stage. Any questionable articles and conflicting opinions during the screening and dual screening process, respectively were discussed by two reviewers. If necessary, a third reviewer was invited to resolve the conflict, and any resulting clarifications to the eligibility criteria were added as notes. None of the reviewers authored any of the articles assessed. The articles that did not meet the criteria at full text were excluded and a list of these with the reasons for exclusion of each article is provided in Additional file 4. To ensure the inter-reviewer consistency, consistency checking was applied at both stages using a random sample of at least 10% of articles.

#### Eligibility criteria

The eligibility criteria for the systematic review are described in Box 1.

**Box 1:** Eligibility criteria

Inclusion criteria		Exclusion criteria
Types of population	Study focuses on human populations in any of the 35 UNESCO biosphere reserves in SEA countries including: Tonle Sap, Cibodas, Komodo, Lore Lindu, Tanjung Putting, Gunung Leuser, Siberut, Giam Siak Kecil-Bukit Batu, Wakatobi, Bromo Tengger Semeru-Arjuno, Taka Bonerate-Kepulauan Selayar, Belambangan, Berbak-Sembilang, Betung Kerihun Danau Sentarum Kapuas Hulu, Rinjani Lombok, Tasik Chini, Crocker Range, Inlay Lake, Indawgyi, Puerto Galera, Palawan, Albay, Sakaerat, Hauy Tak Teak, Mae Sa-Kog Ma, Ranong, Can Gio Mangrove, Dong Nai, Cat Ba, Red River Delta, Kien Giang, Western Nghe An, Mui Ca Mau, Cu Lao Cham-Hoi An, Langbiang	Study focuses on human populations outside the UNESCO biosphere reserves or outside SEA
Types of intervention	Study involves activities/programs/policies* *The activities/programs/policies undertaken must align with the stated functions of UNESCO biosphere reserves, having the aim of one or more of the following: ● Conservation of biodiversity and cultural diversity ● Economic development that is socio-culturally and environmentally sustainable ● Logistic support, underpinning development through research, monitoring, education, and training We will include studies which investigate one or more levels of management within UNESCO reserves, e.g., core zone, buffer zone or transition zone	Study does not involve activities/programs/policies aligned with the stated functions of UNESCO biosphere reserves (see opposite)
Types of comparator	Where present, an eligible comparator is the same site before activities undertaken, or a site without activities (we will note whether the site was designated as a UNESCO biosphere reserve at the time of the comparator), or a site with activities but outside of a UNESCO biosphere reserve	We will include studies even where there is no comparator
Types of study	Studies containing quantitative data (quantitative studies or mixed studies where quantitative data are reported separately)	Qualitative Mixed studies (qualitative and quantitative data are combined and results are not separated for reporting) Theoretical articles, commentary and review papers
Types of outcome	Study focuses on one or more following outcome categories, which are established in the systematic map by Eales et al. [14]: Economic living standard: income, employment, employment opportunities, wealth/poverty, savings, payments, loans Material living standard: access to and availability of food, fibre, fuel and basic infrastructure (electricity, water, telecommunications and transportation), provision of shelter, assets owned (e.g., television) Health: Physical health, mental health, balanced nutrition, longevity/life expectancy, maternal health, infant and child health, birth control provisioning, access to health care (antibiotics, transplants), occurrence of diseases, public health infrastructure (e.g., disease prevention, mental health support) Education: Education infrastructure (access to school, access to training, quality of education, classroom sizes, curriculum relevance and up to date); informal education (transfer of knowledge and skills includes livelihood skills, traditional knowledge and skills); formal education (degrees awarded, students enrolled) Social relations: Interactions between individuals, within and/or between groups (communities, stakeholders, ethnic groups, gender); degree/frequency of conflict, strength of relationships and connectedness, ability to work together, ability to communicate, engage in debate, trust and help others Security and safety: Physical security (personal safety and security), security of access to resources; human rights; vulnerability, personal and community resilience and adaptive capacity Governance: Structures and processes for decision making including both formal and informal rules; includes participation and control in decision making, accountability, justice, transparency of governance Subjective wellbeing: Measures of happiness, Measure of quality of life, Measure of personal satisfaction supported by some value of ecosystem(s) and/or resources Culture and spirituality: Cultural, societal and traditional values of natural resources and nature to the community; sense of home or belonging; cultural identity and heritage spiritual or religious beliefs and/or values Freedom of choice and action: Ability to pursue what you value doing and being; Freedom from norms e.g., gender expectations; Freedom of expression of opinion/beliefs	Study does not measure any socio-economic outcomes
Language	Studies published in English and any other languages within the capability of the review team	Studies published in languages outside the capacity of the review team

### Study validity assessment

We adapted the checklist for quasi-experimental^4^ studies by the Joanna Briggs Institute to assess the validity of selected studies in our review, detailed in Additional file 6 and Box 2. The adaptations were made to better fit the study designs and represent the types of bias we were likely to encounter in this systematic review. We included key aspects of internal validity, for example, whether a study used appropriate methods to detect changes in the outcome. For each of the six criteria, responses are “yes”, “mostly”, “partly”, “no”, “unclear” or “n/a”. The overall validity of each study was classified with an internal validity rating of: High, Moderate, Low and Unclear. Where at least four “yes”, overall validity was “High”. Where at least 3 “unclear”, overall validity was “Unclear”. Where there were either at least two “no” or one “no”, the impact on validity was severe [11], overall validity was “Low”. If none of the above applied, a study was “Moderate”. Because there is a certain degree of subjectivity involved in assigning these ratings, we also provide a validity summary statement to describe the nuances of each of the studies’ validity, which we found valuable when undertaking the narrative synthesis. This validity summary statement reports the main reasons for validity concerns or high validity within a study. Low and Unclear validity studies will not be excluded from our review, but we narratively explore the impact of including or excluding these studies in the narrative synthesis. We also assessed the external validity, i.e., whether the PICO elements of the study reflected the PICO elements of our review. Because of the intrinsic difference between internal and external validity, we report external validity separately, in descriptive statistics, whilst the internal validity is reflected in our internal validity ratings, reported in Table 1 and referred to in the narrative synthesis.

**Table 1:** Characteristics of 16 studies investigating socio-economic impact of activities in UNESCO BRs in Southeast Asia

Article; type of data UNESCO BR, Country	Study design	Population	Intervention category and duration of intervention	Outcome category	Data collection method	Internal validity rating
Bahadur, 2020 Quantitative Tonle Sap, Cambodia	Non-controlled, (cross-sectional)	Households dependent on fish	CBNRM 3 years	Economic living standards Governance Social relations Subjective well-being Material living standards	Survey	High
D'Agnes, 2010 Quantitative and Qualitative Palawan, Philippines	BA	Household in Palawan	CBNRM Health intervention CBNRM & Health intervention At least 4 years	Health Economic living standards	Household survey (not matched pre-post, they were different, deliberately)	High
Dinh, 2010 Quantitative Cat Tien (Dong Nai), Vietnam	CI BACI	Household in biosphere reserve	Resource Use Management 4 years	Economic living standards	Household surveys	Low
Dumlao, 2003 Quantitative and Qualitative Palawan, Philippines	CI	Coastal community	Habitat management 1996 (across 6 years)	Economic living standards Social relations	Interview with structured questions	Moderate
Eriksson, 2019 Quantitative and Qualitative Komodo MPA, Indonesia	CI	Residents	Resource Use Management Since 1980 (BR) Since 2010 in (outside BR)	Economic living standards Education Social relations	Semi-structured interviews	Moderate
Ngoc, 2018 Quantitative Cu Lao Cham, Vietnam	Non-controlled, (cross-sectional)	Longline fishers Gillnet fishers Liftnet fishers Fishing households	Resource Use Management 10 years	Economic living standards Material living standards Economic living standards Governance	Survey	Unclear
Nguyen, 2019 Quantitative and Qualitative Cu Lao Cham, Vietnam	Non-controlled, (cross-sectional)	Local people working in tourism sector	Livelihood intervention NR	Social relations Subjective well-being Economic living standards	Interviews	Low
Palmer, 2014 Quantitative and Qualitative Lore Lindu, Indonesia	Non-controlled, (cross-sectional)	Communities in 3 areas impacted by NGOs	CBNRM years 14 years approx	Governance	Community surveys	Moderate
Pido, 2009 Quantitative Palawan, Philippines	Non-controlled, (cross-sectional)	Local residents	CBNRM 6 years maximum	Material living standards	Individual and household interviews	Low
Richardson, 2018 Quantitative and Qualitative Tonle Sap, Cambodia	CI	Fish farming/former fish farming households	Livelihood intervention 4 years	Material living standards	Household survey	Moderate
Shively, 2001 Quantitative Palawan, Philippines	CI BA	Community (farming)	Livelihood intervention NR	Economic living standards	Survey	Unclear
Sok, 2012 Quantitative Tonle Sap, Cambodia	Non-controlled, (cross-sectional)	Households	CBNRM 8 years approx	Social relations	Survey	Moderate
Torell, 2010 Quantitative Ranong, Thailand	CI Non-controlled, (cross-sectional)	Households	Livelihood intervention NR	Economic living standards Social relations Governance Education	Survey	High
Tran, 2014 Quantitative and Qualitative Red River Delta, Vietnam	CI	Local residents participating in scheme	Livelihood intervention 2006–2011 (funded)	Economic living standards	Survey	Moderate
Tupper, 2015 Quantitative and Qualitative Palawan, Philippines	Non-controlled, (cross-sectional)	Stakeholders for the Calamianes islands MPA	Resource Use Management 2–4 years, depending on the MPA site	Governance Economic living standards	Workshop on perceptions	Moderate
Vong, 2017 Quantitative and Qualitative Tonle Sap, Cambodia	CI	Households	CBNRM 9 years	Material living standards	Household interview, semi-structured questionnaire	Moderate

*BA* before–after; *CI* control-impact, *CBNRM* community-based natural resource management, *MPA* marine protected area, *NGO* non-governmental organization, *NR*  not reported

**Box 2:** Study internal validity assessment

Internal validity criteria	Type of bias addressed
Was the study free of any *baseline* factors that may be associated with both the intervention and outcome of interest (or different participant groups if non-comparative)?	Confounders at baseline/selection bias
Was the study free of any external events or factors during the study that could influence the outcome of interest?	Confounding
Were the intervention (and comparator, if present) clearly defined, at an appropriate temporal and spatial scale, and adhered to as intended?	Appropriate intervention/comparator
Was an appropriate method used to measure the outcome across all study groups?	Detection bias
Were all data reported, or was the study clear of any systematic differences between study groups in the number of missing data?	Attrition bias
Were all outcome measurements reported appropriately?	Outcome reporting bias

Each study was assessed by at least one of two reviewers, and a second reviewer checked each of the study assessments. Any discrepancies were discussed, and amendments made after arriving at a joint decision.

### Data coding and extraction strategy

An Excel spreadsheet for data extraction (meta-data and quantitative data) was used to collate information from each study including: study site/area/year of Biosphere Reserve designation, study design, details of the population and intervention (including duration) and outcome details including data collection method and duration of collection. Where quantitative data was extracted, the location of the data within the article was recorded (e.g., table, figure or page numbers), and all raw data extracted as presented by authors. There were no instances where data needed to be extracted from graphs or where there was a discrepancy between data in the text and in tables or figures. We contacted authors for any key missing information, and any responses providing useful detail were added into the data extraction.

Data extraction was conducted by at least one of two reviewers (JE and NPT). Six of the 16 studies were independently data extracted and cross-checked to address potential disagreements and inconsistencies in data extraction. Data extraction forms were adapted, and completion notes expanded on to provide further clarity. The remaining ten studies were data extracted independently by one reviewer, followed by a further round of checking by one of the two reviewers. The full data extraction spreadsheets are presented in Additional file 7.

### Potential effect modifiers/reasons for heterogeneity

After consultation with Marine Science and Social Science researchers within the Blue Communities Programme and based on previous research articles in this topic area, we compiled a (non-exhaustive) list of factors that may influence the strength of effect. These included: geographical location, the area of UNESCO biosphere reserve, year of designation, governance (leadership, building partnerships, government and stakeholder commitment, support and on-going support), participation and collaboration of local community, public, private stakeholders and NGOs, and characteristics of landscape and zonation.

Some of the effect modifiers we identified in the protocol were difficult to fully examine during narrative synthesis, due to a lack of information provided by studies, or due to a lack of connection between a particular effect modifier and the outcome. These included: funding and human resources of the reserves (staff experience, knowledge and availability); management plans and vision; monitoring and evaluation frequency and indicators; research integration (connection to research institutes) and land use in the surrounding area before and during the designation.

### Data synthesis and presentation

We present our review findings, first the results of the search and descriptive statistics, followed by a narrative synthesis, which explores the evidence base, grouping studies by the outcomes investigated. We explore effect modifiers and compare the activities with both positive and negative impacts of UNESCO biosphere reserves on socio-economic wellbeing. We were unable to undertake a quantitative synthesis, due to heterogeneity between studies in design and focus.

## Results

### Search and screening results

**Fig. 1:**
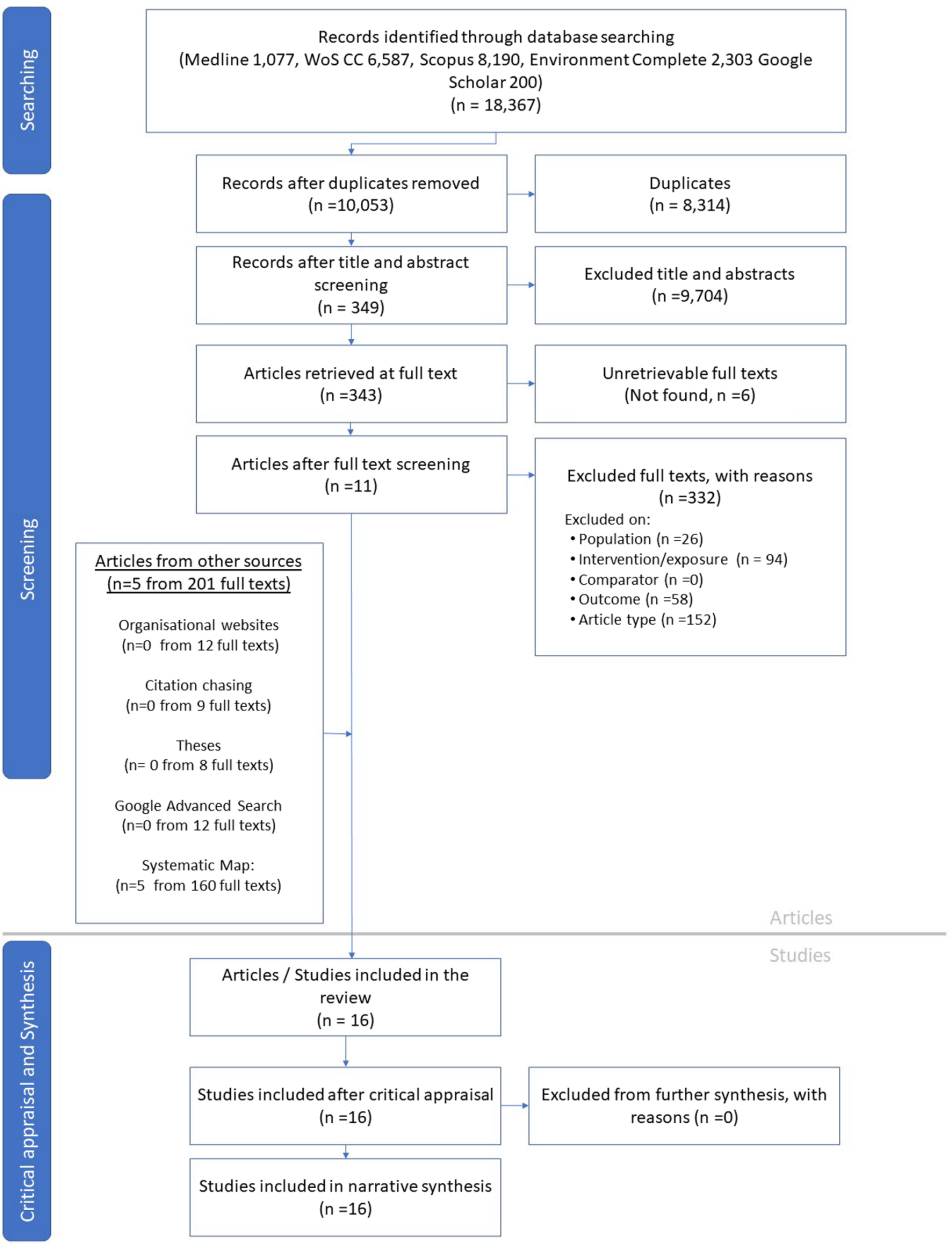
Flow chart presenting the movement of articles through this review

We provide a ROSES flow chart (Fig. 1) showing the number of included articles for each stage, in line with CEE guidance. In total, we assessed 10,053 records at title and abstract from database searches and Google Scholar, of which 9704 were excluded, when assessed against the eligibility criteria. Though this number of excluded studies seems large, it is demonstrative of a comprehensive and sensitive search strategy, which is a key aim of a systematic review search. We deliberately ensured that our search was wide in scope, by using only the name of each BR, together with (“AND”) the keywords “biosphere reserve”. We wanted to minimise the likelihood of potentially relevant studies not being retrieved by our search, and made a careful judgment, using search term scoping to ensure we had the resources to assess the likely number of hits retrieved by our sensitive search. The number retrieved is in line with other systematic review search strategies (e.g., Eales et al. [13], retrieved 12,971 unique records) and is accordingly fewer than for systematic maps (e.g., Eales et a. [14] retrieved 42,894 unique records, Short et al. 2021 retrieved 15,680 unique records). We were able to retrieve 343 of the 349 records included based on their title and abstract. Together with 201 articles from supplementary searching, this totalled 544 articles assessed based on their full text. Of these, 528 were deemed not relevant, and reasons for exclusion are provided in Additional file 4.

The results of consistency checking are given in Additional file 5. At title and abstract stage, 1258 (12.5%) of the 10,053 articles from database searches were screened by at least 2 reviewers. Agreement was either 100% or Kappa “Substantial”, between pairs of reviewers. At full text screening, 58 articles (17%) were double screened from a total of 349 that were included at title and abstract stage from the database searches. Agreement levels were all over 90% and there was “Substantial” agreement in Kappa scores between pairs of reviewers. 16 articles, corresponding to 16 studies were included in our review, listed in Table 1. Additional file 7 presents the data extracted for this review. Table 1 presents key information from each study, whilst Table 2 focuses on the intervention and outcome combinations from each study.

**Table 2:** Intervention, comparator, outcome details and summary of results from the 16 articles included in this review

Article validity	Intervention description	Comparator used	Outcome description	Summary of relevant results
Bahadur, 2020 High	Community fishing policy, abolition of lots	n/a	*Households dependent on fish:* *% decreased/increased/same* Profit from fishing Conflict with law enforcement Conflict with other fishers Fear of getting into trouble Happiness	*After 3 years of fishing legislation in Tonle Sap:* Profit made from fishing did not change significantly (46.5/38.2/15.35%; n 484) Conflict with law enforcement has decreased (77.4/7.9/14.7%; n 469) Conflict with other fishers significantly decreased p < 0.1 (78.1/5.1/16.8%; n 489) Fear of getting into trouble significantly decreased p < 0.05 (49.3/28.6/22.1%; n 465) People's general happiness improved (20.1/35.1/44.8%; n 467)
			*% yes/no* Access to fish better? % yes/no Livelihood income better? % yes/no	Access to fish improved significantly p < 0.05 (75/25%; n 428) Livelihood and income did not change significantly (47.9/52.1% n 422)
D'Agnes, 2010 High	CBNRM: marine/mangrove protected areas, reforestation, enforcement, peer education and behaviour change communication; prohibiting dynamite and cyanide use in fishing RH: family planning, non-clinical contraceptives, peer education and behaviour change communication, improved access to RH services CBNRM + RH integrated as one package	Before CBNRM Before RH Before CBNRM + RH	*Proportion of households 4 years after intervention:* Youth contraceptive use at first sexual experience	*Youth contraceptive use at first sexual experience (n* = *400 each intervention):* No sig difference after CBNRM intervention No sig difference after RH intervention Sig increase after CBNRM + RH intervention
		Before CBNRM Before RH Before CBNRM + RH	Young (15–24) males sexually active	*Proportion of young males sexually active (n* = *400 each intervention):* No sig difference after CBNRM intervention No sig difference after RH intervention Sig decrease after CBNRM + RH intervention
		Before CBNRM Before RH Before CBNRM + RH	Households dependent on fishing	*Proportion of households dependent on fishing (n* = *400 each intervention):* No sig difference after CBNRM intervention No sig difference after RH intervention Sig decrease after CBNRM + RH intervention
Dinh, 2010 Low	Core zone, ban on exploitation Buffer zone Transition zone	n/a	% change in gross annual income from forest per capita between 1998 and 2005	Change in % gross annual income from forest per occupant between 1998 and 2005 was highest in core (83%) compared to buffer (27%) and transition (3%) and shows local people kept using forest resources despite ban in Core zone, Cat Tien 1998–2005. n for the three zones was 18; 51; 18 respectively
	*After designation:* Core zone Buffer zone Transition zone	*Before designation:* Core zone Buffer zone Transition zone	Gross annual income from forest—1000 Vietnamese Dong/occupant/year	Gross annual income from the forest was similar across zones (though higher in core than buffer and transition, 1840 vs 1570 vs 1470) before 1998 designation of Cat Tien BR. By 2005, it had increased sevenfold in core, threefold in buffer and slightly increased in the transition (12,580 vs 4540 vs 1760). n for the three zones was 18; 51; 18 respectively
	*After designation:* Core zone Buffer zone Transition zone	*Before designation:* Core zone Buffer zone Transition zone	% of household income from forest resources	1998–2005 the % of income from forest increased most in core zone (from 67 to 97%, t = − 5.212, df 17, p < 0.001), then in buffer (from 58 to 64%, t = − 2.031, df 50, p < 0.05), while it dropped in transition zone (from 45 to 19%, NS). n for the three zones was 18; 51; 18 respectively
Dumlao, 2003 Moderate	Mangrove rehabilitation project	Non members of the mangrove rehabilitation project	*% local people reporting:* Increased family income	*Comparing perspectives of members of a 6-year mangrove rehabilitation project and local non-members (n* = *15 in each group):* 73% of members had uplift in family income through financial support by the project, whilst none of the non-members did. However, 23% of members said their income had decreased due to mangrove planting (e.g. time spent planting has low monetary return)
			Tensions in local social relations	All respondents in both groups experienced project-related social tensions e.g. (misunderstandings, boundary and area conflicts and lack of cooperation). This is likely due to/exacerbated by project implementers not providing solutions or guidance for these problems
Eriksson, 2019 Moderate	Shark and manta ray conservation—fishing zone restrictions, fishing gear restrictions	Non-BR with intervention	Effect of conservation on economic income % increase/decrease/no mention	*Comparing Komodo National Park in BR (n* = *30) and Nusa Penida outside BR (n* = *14):* Komodo, 63% of the 30 respondents experienced a negative effect on income from Park (10% increase in income, 27% did not mention): the zoning system complicated access to fishing grounds, and they had to invest in new fishing gear Nusa Penida: 43% of 14 respondents had increased income and none experienced a negative effect (57% did not mention). Many said livelihoods depend on the region being protected, especially those working part-time in tourism
			Knowledge of conservation rules % responding high/low/no mention	Komodo: 43% had a high degree of knowledge of conservation rules and 40% a low degree (17% did not mention). Also many complained of poor access to information about regulations Nusa Penida: 72% had a high degree of this knowledge, 14% a low degree and 14% did not mention
			Relationship with conservation authorities % responding good/bad/no mention	Komodo: relations between fishing communities and conservation authorities and rangers were poor according to 67%, only 20% mentioned good relations, 13% did not mention. Some said park rangers were threatening. Lack of dialogue for the zoning system led to past disputes Nusa Penida: a majority had good opinion of MPA management and boat patrols (86%), seeing them as friendly, respectful, and trustworthy. They said fishing had improved since protection from destructive fishing gear. None said there was a bad relationship, 14% did not mention
			Access to alternative livelihoods % responding good/poor/no mention	Komodo: only 23% had access to alternative livelihoods, most said access was poor (67%), 10% did not mention. The main obstacle being a lack of access to financial support to start businesses such as shops or street kitchens Nusa Penida: access to additional source of income was perceived as good (64%) especially tourism which was used by half of interviewees. None described it as poor, and 36% did not mention
Ngoc, 2018 Unclear	Core zone, with fishing ban	n/a	Litres extra fuel usage per trip	Since the MPA ban on fishing, across 135 fishers, more litres of fuel were used to access fishing grounds: liftnet fishers worst affected, then longline, then gillnet (x̄ 4.2 L per trip vs 3.8 and 1.1)
	MPA		*Fishing households' (Likert scale 1–5) perceived impact of MPA on:* Food security Tourism jobs and additional income Participate in creating/managing MPA regulation	*Across 135 fishers, perceived impact of MPA after 10 years' implementation in Cu Lao Cham:* Food security: mean closest to "somewhat agree" (x̄ 3.95) Tourism jobs and extra income: closest to "somewhat agree" (x̄ 3.79) Participate in MPA regulation: between "somewhat disagree" and "neither agree nor disagree" (x̄ 2.6)
Nguyen, 2019 Low	Initiatives to aid local people participation in tourism services: Training courses for local guides and motorbike taxi groups	n/a	*% tourism workers reporting conflict:* Between local stakeholders and tour operators Between freelance guides and tour guides Among local tourism stakeholders	*Of 41 local respondents working in tourism in Cu Lao Cham, regarding tourism and initiatives by CLC Tourism Management board:* 29% said it caused social conflicts between local tourism stakeholders and tour operators, 10% said it caused conflict between freelance guides and tour guides, and 12% said it cause conflict among local tourism stakeholders
			Perceived quality of life % improved a lot/improved a little/no change	68% perceived it improved quality of life a lot, 15% improved a little, and 17% no change
			% reporting jobs provided: for poor/for women	90% said tourism provided jobs for the poor, and 90% said it provided jobs for women
Palmer, 2014 Moderate	NGOs supporting indigenous rights to resource extraction	n/a	*Participated in negotiation, YTM/YJ site, mean of responses (1* = *yes, 0* = *no):* All household heads Some household heads	*In Lore Lindu, after 14 years of NGO support for indigenous rights by YTM/YJ, negotiations for comanagement involved:* Low participation of ALL household heads (x̄ 0.29, SD 0.46, n 7) Moderate participation of SOME household heads (x̄ 0.62, SD 0.50, n 7)
	The Nature Conservancy (TNC) focus on biodiversity conservation	n/a	*Participated in negotiation, TNC site, mean of responses (1* = *yes, 0* = *no):* All household heads Some household heads	*In Lore Lindu, after 14 years of NGO support for indigenous rights by TNC site, negotiations for comanagement involved:* No participation of ALL household heads (x̄ 0, n 21) High participation of SOME household heads (x̄ 0.86 SD 0.38, n 21)
Pido, 2009 Low	Locally managed MPA	n/a	% low/middle/high Material lifestyle local house construction materials	For 135 households within MPA in Palawan, using household building materials as a proxy, material style of life measures were: low 30.4%; middle 36.3%; and high 33.3%
Richardson, 2018 Moderatre	Small-scale aquaculture pond management program (Cambodia HARVEST Aquaculture and Fisheries Program)	No intervention (people who discontinued)	% fish-farming households that sometimes cannot afford to feed family	In household survey at Tonle Sap lake in 2015, locals who continued on aquaculture program (n = 324) had fewer problems feeding their families than those who discontinued, n = 112, (23% vs 40%) but the difference was not significant (z score = 1.381 p = 0.1673)
Shively, 2001 Unclear	Farms on biosphere reserve with irrigation	Biosphere reserve site with no irrigation	Total income (Pesos) of farm household	A significant difference (only at the 90% level) in income between irrigated (x̄ 104,128; n 46) and rain-fed (x̄ 108,867; n 35) lowland rice farms in Palawan
	Irrigation	Before irrigation	Days of employment for farm labourers	More days of employment available on upland farms after irrigation systems added than before (x̄ 44 vs 18, n unknown), but not significant at 95% level. Unknown duration of irrigation
Sok, 2012 Moderate	Community fishing project	n/a	*Participation in fisheries project (% women/men): *community meetings group discussions workshops	Across 301 respondents, for a fisheries project, there was significantly different participation in: community meetings: odds ratio of 3.8 at p < 0.05 (46.3/75.2%) group discussions: odds ratio of > 3.5 at p < 0.05 (38.4/67.9%) workshops: odds ratio of 2.1, at p < 0.05 (48.8/64.2%) Other activities not significantly different between men and women included training, cross visits, whiteboard information, bulletins, advocacy campaigns, and commune investment plan development
Torell, 2010 High	Micro-credit and new enterprises to diversify livelihoods by resource management and small scale infrastructure in five communities severely affected by 2004 tsunami	Non project beneficiaries	Number of livelihoods per household	*Household survey of micro-credit intervention in Thailand:* Number of livelihoods per household showed no significant difference between beneficiaries (x̄ 1.92; SD 0.932; n 226) and non-beneficiaries (x̄ 2.04; SD 0.979; n 228); t = 1.328, df = 452, p > 0.1
		n/a	*Household response for:* Create stronger social ties Improve coordination with local government Develop my business skills	*% of micro-credit beneficiaries (n* = *226) who chose 4 ("agree") on Likert scale 1–5 for:* Creating stronger social ties: 73% Improving coordination with local governments: 51% Developing business skills: 67%
Tran, 2014 Moderate	Giao Xuan ecotourism project with hosting to develop the skills of community members (especially poor women and fishermen) in conservation-based eco-business	Non-host families within the ecotourism project	USD monthly income of resident families participating in scheme	In a capacity building ecotourism project over 5 years with 11 host families involved in the project, ecotourism income $47 per month, twice the average for non-hosting project members (n = unclear, possibly 6). The hosts' ecotourism income was twice as much as from wet rice cultivation
Tupper, 2015 Moderate	Sagrada-Bogtong Marine Reserve Decalve Strict Protection Zone (Bintuan-Sangat Marine Park) Bugor-Sand Island MPA	n/a	*MPA stakeholders' ratings of:* "Existence of a decision-making & management body" "Existence and adequacy of enabling legislation" "Degree of interaction between managers & stakeholders"	*Household survey of 28 Calamianes Islands MPA stakeholders:* Across all three sites, adequacy of decision-making & management body, and enabling legislation, and interaction between managers and stakeholders, were positive except for the last which was negative only in Sagrada-Bogtong,
	Sagrada-Bogtong Marine Reserve Decalve Strict Protection Zone (Bintuan-Sangat Marine Park) Bugor-Sand Island MPA		"Perceptions of local resource harvest" "Number and nature of markets"	Across all three sites, perceptions of local resource harvest were negative, e, and perceptions of the number and nature of markets were positive
Vong, 2017 Moderate	CBNRM in Chivieng	No CBNRM	Household expenditure USD on food, non-food and non-timber forest products	Even though CBNRM did not positively affect the household consumption in Chivieng community as a whole (possibly due to the impact of migrant fishers), in the CBNRM community, households who fished only inside the community boundary, had higher household consumption than those fishing within boundary in the non CBNRM community (x̄ = 56.4 Range (15.57–138); n 156 vs x̄ = 33.95 (12.51–110); n 192, NS

### Descriptive statistics

**Fig. 2:**
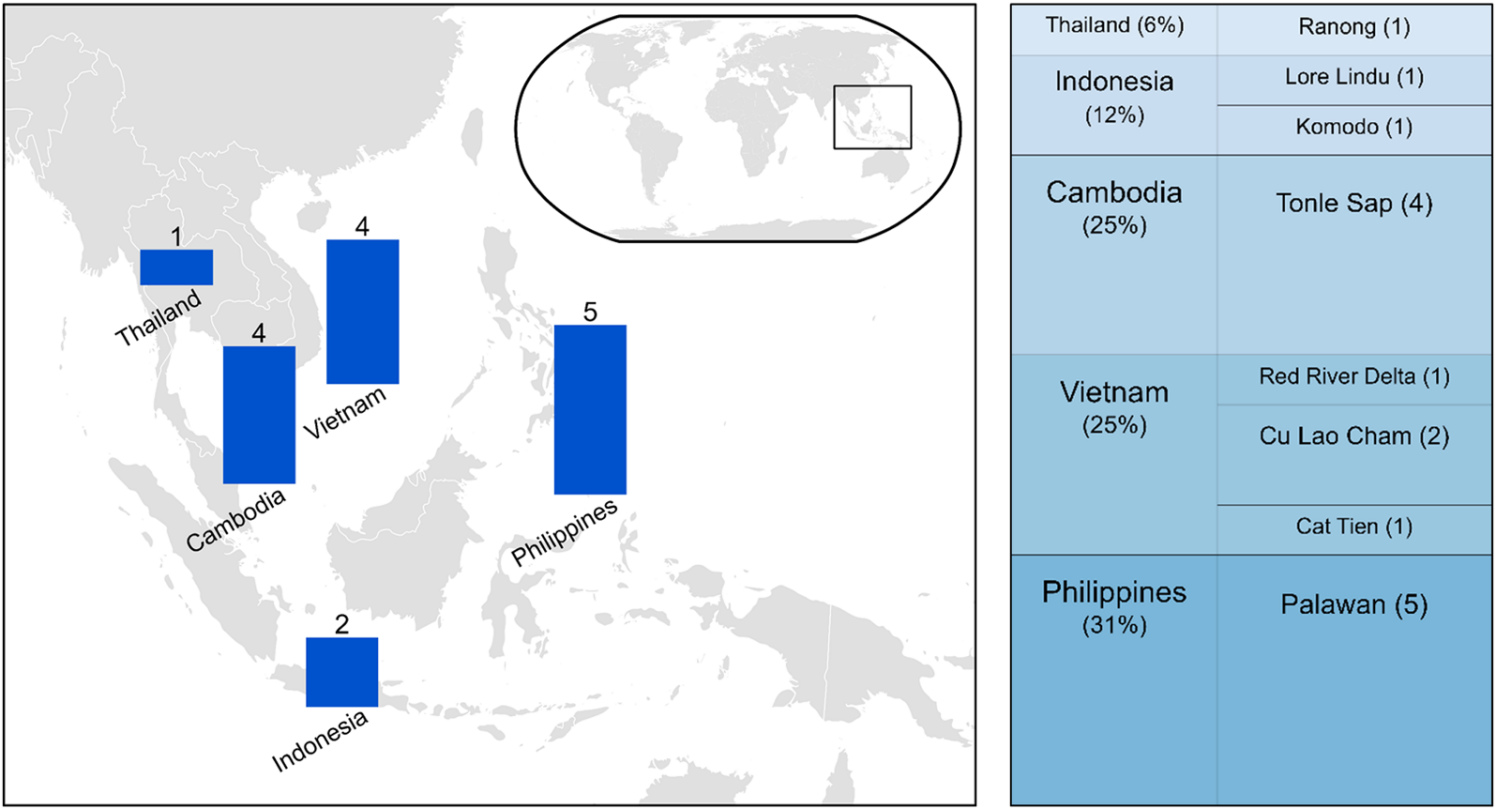
Map showing number of studies included in this review by country, and stacked bar showing the proportion of studies undertaken by country (%) and UNESCO biosphere reserve (number of studies) featured in the 16 studies

**Fig. 3:**
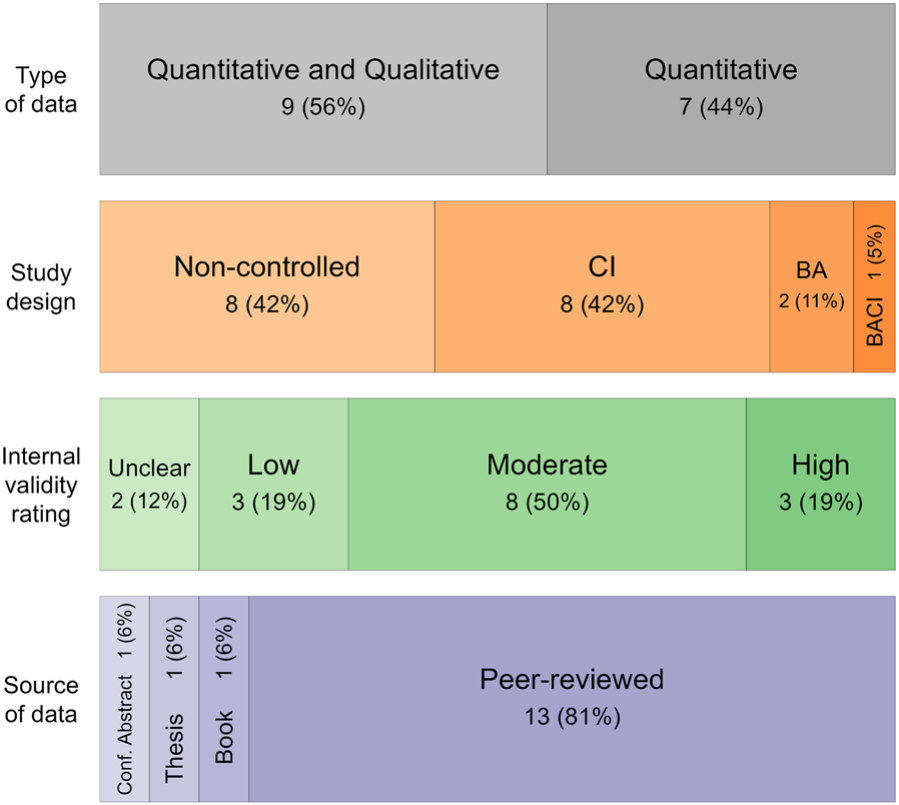
Data type, study design, internal validity rating and source of article for the 16 included studies in this systematic review. Some articles reported data arising from multiple study designs, hence the total number of study designs is greater than 16

We present the descriptive statistics in Table 1, and in Figs. 2 and 3.

#### Article location, article type, data type, study design and validity

Of the 16 studies, 5 were in the Philippines (all in Palawan), 4 in Vietnam (Cat Tien/Dong Nai, Cu Lao Cham and Red River Delta), 4 in Cambodia (all in Tonle Sap), 2 in Indonesia (Lore Lindu and Komodo MPA) and 1 in Thailand (Ranong). This does not reflect the range of UNESCO BR sites in the region, of which there are 35 (we found studies focusing on 8), and we found no relevant studies from Malaysia or Myanmar. An interactive map of the studies can be accessed at https://unesco-br-sr.github.io/

Thirteen (81%) were peer reviewed articles, the remaining three were a book article, a thesis and a conference abstract. Nine studies contained qualitative as well as the quantitative data that was relevant to this review, the remaining 7 studies were focused only on quantitative data. Articles sometimes made more than one comparison with their datasets, hence, the evidence base comprised: 8 non-controlled (cross-sectional) studies; 8 control-impact (CI) studies (i.e., a spatial comparator, or a population group not receiving the intervention); 2 before-after (BA) studies and one before-after-control-impact (BACI) study. Some of the non-controlled studies reported differences between population subgroups (e.g. type of fisher [24], or type of intervention (e.g. NGO focus [27]. Of the 16 studies, 3 were assigned overall high validity, 8 overall moderate validity, 3 overall low validity and 2 studies did not provide sufficient information for categorisation (unclear validity) Table 1 and Fig. 3. The details of the validity assessment are in Additional file 6. The external validity (whether studies’ PICO aligned to the PICO of this review) varied across studies between yes, mostly, and partly. Seven studies were categorised as partly (Richardson, Shively, Sok, Palmer, Nguyen, Pido and Tupper) for external validity, i.e., the question “Do the PICO elements of the study match the PICO elements of this review?”.

There were no articles first authored by the same author, and only one author who contributed to two articles [28] and [38], which shared none of the same interventions or outcomes and were undertaken in different sites, though in the same BR, Palawan. There were no instances where institutions or research centres were predominant in the evidence base.

#### Population, interventions, comparators and outcomes

**Fig. 4:**
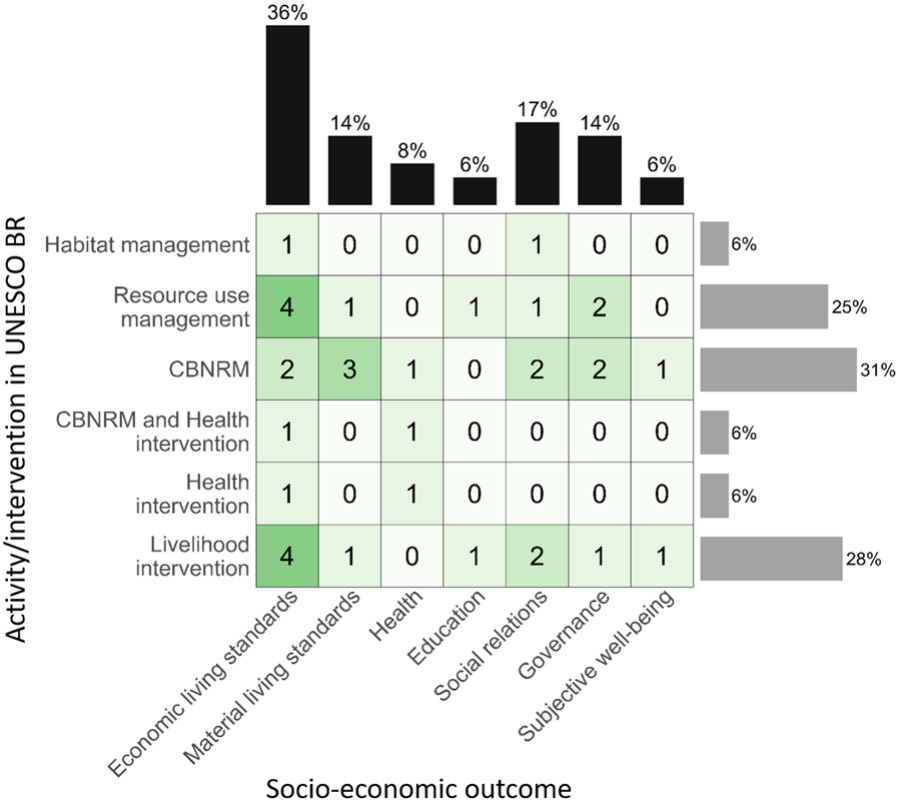
Heatmap showing where interventions/activities in BRs were undertaken, and socio-economic outcomes were reported by the 16 articles in this systematic review. Values within boxes indicate the number of studies. Bars show the percentage representation of the activities/interventions and the socio-economic outcomes in this systematic review

The matrix in Fig. 4 shows a heatmap of the number of studies which investigated the outcomes and interventions (by categories). The numbers in the heatmap are higher than the number of articles, because some studies reported multiple interventions or outcomes. Six of the studies focused on Community-Based Natural Resource Management (CBNRM), 5 on livelihood interventions, 4 on resource use management and one was a habitat management intervention. In terms of outcomes, 11 studies measured economic living standards (one of these studies measured this with two different measures), 4 measured material living standards. Governance was an outcome for 5 studies. 6 studies measured social relations and subjective wellbeing was measured in two studies. Education was an outcome measure in two studies, and health was only measured by one study.

Table 1 summarises population characteristics (further details in Additional file 7), Table 2 summarises characteristics of the intervention, comparator and outcomes. All study populations were local (as defined in the eligibility criteria), and were typically households, or residents. Occupation where reported, was typically fishers, or those dependent on fish (e.g., Bahadur et al. [2]), but was sometimes those with additional or multiple jobs (e.g., Nguyen [26], farming communities (e.g., Shively and Martinez [32] or those participating in the intervention (e.g., [37, 38] gathered data from the MPA’s stakeholders). One study, [24], reported data separately for different types of fisher. Where reported, the age of the study population was adults, or all ages [11], Additional file 7. The duration of intervention varied widely, and was reported in different ways by authors, ranging from 2 years for some MPA interventions in the study by Ngoc [38], to “since 1980” for one of the shark and manta ray management interventions in the Eriksson 2019 study.

Comparators were used in different ways by different studies. Two studies reported a “before” comparator, i.e., the same site before intervention, before-after studies [10, 32]. Of the control-impact studies, three studies used a comparative site, i.e., a site/area without intervention [11, 32, 39]. Four other studies compared with local people who were not involved in an intervention/project [12, 29, 36, 37]. One study [15] used a comparator which was a site with the intervention but outside of a UNESCO biosphere reserve. One study [11] reported comparators of both before intervention, and control sites (before-after, control-impact studies). Two studies also investigated differences between different types of intervention [27], reported two types of CBNRM supported by different NGOs, and D’Agnes et al. [10] reported data from three types of intervention: CBNRM, Reproductive health and CBNRM with Reproductive health.

#### Evidence gaps and relations with the Seville strategy aims

Of the 10 outcome categories identified in the protocol, 7 were found in the studies included in this synthesis, namely economic living standards, material living standards, governance & empowerment, health, social relations, subjective wellbeing and education. Those outcomes not investigated by studies in our review were: freedom of choice and action, security and safety, and culture and spirituality. This evidence gap may demonstrate the concentration of researchers on outcomes that are quantifiable (the three missing outcome categories are typically measured with qualitative methods), and a reflection of the fact that we included only quantitative and not qualitative data in this review. Of the 7 outcome categories covered by studies in our review, economic living standards was the most often reported (14% of studies), which echoes the finding from the systematic map in [14]. Least reported outcomes were education (6%), health (8%) and subjective wellbeing (6%). Social relations (17%) and governance (14%) were as well-documented as material living standards (14%), a reflection of the recognition of these outcomes as important considerations when implementing interventions that affect residents of biosphere reserves.

The intervention categories in the heatmap in Fig. 4 are not intended to be used alone as a tool to identify gaps in the wider evidence base, because these categories were determined by those we found in studies in our review. We chose this approach, rather than creating a heatmap matrix with the three Seville strategy aims as the intervention categories, because interventions as described by several studies addressed multiple aims. Thus, we use the heatmap as a tool to narratively describe the intervention gaps in relation to the Seville strategy aims. This was a challenging exercise because many activities addressed multiple goals, though we do highlight two key gaps. There was a lack of studies that specifically focused on the “conservation of cultural diversity”, although one study [27], did investigate a CBNRM supported by NGOs which aimed to support indigenous rights to resource extraction. Also missing were studies investigating the Seville strategy aim of “logistic support, underpinning development through research, monitoring, education and training”, although one study did include activities that supported tourism operators [26].

“Conservation of biodiversity” was a Seville strategy aim which was generally well addressed through combinations of CBNRM and resource use management. We found that habitat management alone was not commonly evaluated (only reported in one study, for mangrove rehabilitation [12]. This was not surprising since UNESCO BRs offer opportunities for integrating management of resources alongside habitat, and for involving communities in management, both of which were better represented (in 9 and 11 cases, respectively). In the process of managing BRs, many actions are implemented and vary from country to country. The common goals of BRs are to protect the core area, to find effective solutions to manage and exploit the resources of the BR, in which, CBNRM is a method that has been popularly applied in Vietnam and other places with natural resources, including non-BRs too. However, holding the title of a UNESCO BR, the local state agencies themselves and local people are aiming for a common goal, advantage in finding a common voice between state agencies and their communities. There were a limited number of studies focusing on health interventions, perhaps not surprising, since this is not a specific goal of UNESCO BRs. Biosphere reserves are ‘learning places for sustainable development’. They are sites for testing interdisciplinary approaches to understanding and managing changes in socio-ecological systems, including conflict prevention and management of biodiversity. However, there have been limited interdisciplinary and transdisciplinary studies in the BRs, especially studies on human health of local people in the BRs. Our research results partially pointed out that researchers, authorities should pay more attention on these issues to give a whole picture of BRs and its human-environment relationships. We identified 10 instances where studies reported on livelihood interventions. These included micro-credit [36], support for farming [32] and aquaculture [29] and tourism support [26]. However, it was not always clear that the livelihood support was in line with the Seville strategy aim of “Economic development that is socio-culturally *and* environmentally sustainable”.

Linking the evidence gaps we have identified in this review with the known activities in UNESCO BRs will highlight if and where follow up monitoring needs to be undertaken and made available to evaluate the impacts of activities in UNESCO BRs. Our research results have shown that more interdisciplinary and transdisciplinary research is needed to determine the effectiveness of this model, because in fact some (45 sites at 2018 UNESCO ICC meeting) BRs no longer meet the commitments and standards to maintain their designation^5^,^6^ and were accordingly redacted as UNESCO BRs.

### Narrative synthesis

**Fig. 5:**
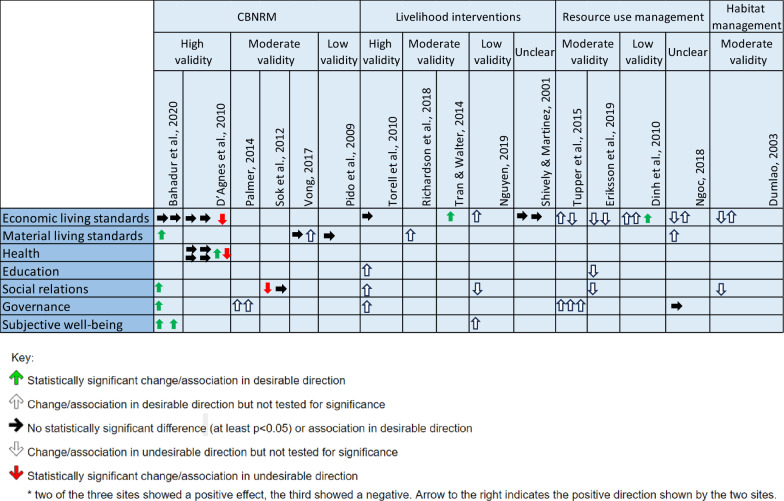
Visual summary of impact of interventions across the 16 studies, separated by validity, each arrow represents a single outcome measure

Figure 5 provides a simplified, visual summary of the impact of the interventions, presented across the outcome category (rows) and shown by study (columns). Because studies sometimes measured outcomes using multiple measures, there are more associations than the 36 instances shown in Fig. 4.

#### Economic living standards

Eleven studies in our review reported economic outcomes. Three studies were assigned overall high validity [2, 10, 36], four overall moderate validity [12, 15, 37, 38], two overall low validity [11, 26] and two overall unclear [24, 32]. Six studies used surveys with questionnaires, four studies used interviews with both semi-structured and structured questions, one study combined both questionnaire and stakeholder consultation workshops.

Economic living standards include any outcomes related to income, employment, employment opportunities, wealth/poverty, savings, payments or loans. Of the 11 studies reporting results on economic living standards, six studies focused on income and other elements indirectly impacting income (e.g., extra fuel usage for fishing trips, profit from fishing), one study focused on dependence on fishing, two studies reported results on livelihoods, two studies reported results on employment and one study reported results on resource harvest and market. The impact of these interventions is summarised in the first row of Fig. 5.

##### Income

Four studies (two moderate, one unclear and one low validity) reported generally positive impacts, one moderate validity study reported negative impacts and two studies (high and unclear validity) reported no significant impacts on income.

##### Positive impact

Ngoc [24], Dumlao [12], Tran and Walter [37] and Dinh et al. [11] reported generally positive impacts on income of local people living in BRs, though none of the studies reported/tested whether the differences were significant. A non-comparative survey by Ngoc [24], unclear validity, found that after 10 years of Cu Lao Cham MPA (Vietnam) being in place, most fishing households “somewhat agreed” that the MPA had generated tourism jobs and extra income. However, this study also indicated that since the MPA ban on fishing, all types of fishers used more litres of fuel to access fishing grounds that were further away because of zoning. A comparative study by Tran and Walter [37], moderate validity, found that a community-based ecotourism (CBET) project in Red River Delta BR (Vietnam), helped CBET families who hosted tourists generate an ecotourism income $47 per month, twice the average for non-hosting CBET families. The hosts' ecotourism income was twice as much as from wet rice cultivation. The comparative study by Dumlao [12], moderate validity, reported different impacts of a 6-year mangrove rehabilitation project on income. On one hand, 73% of project members reported that their family income increased due to financial support by the project, whilst none of the non-members reported any increase. On the other hand, 23% of members said that their income had decreased due to mangrove planting (time spent planting has low monetary return).

A household survey by Dinh et al. [11] with low validity, in Cat Tien BR (Vietnam) investigated income change before and after BR designation (1998). It found that the change in gross annual income from forest per occupant per year by BR zone shows local people kept using forest resources despite the ban in the core zone. The gross annual income was improved after the designation. Specifically, it was similar across zones (though higher in core zone) before 1998 designation of Cat Tien BR. By 2005, it had increased sevenfold in the core zone, and threefold in the buffer zone. In the period of 1998–2005, the percentage of income from forest increased most in core zone (from 67 to 97%, significant at p < 0.001), then in buffer (from 58 to 64%, significant at p < 0.05), while it dropped in transition zone (from 45 to 19%, NS). The study was rated as low validity mainly because of several external factors that were likely to have heavily impacted the outcome (subsidies and aid awarded during the study period).

##### Negative impact

One comparative study by Eriksson et al. [15], moderate validity, reported a negative effect of shark and manta ray conservation efforts on economic income in a UNESCO BR. In Komodo National Park, 63% of interviewed people experienced negative economic effects from the park, because the zoning system caused complicated access to fishing grounds, and they had to invest in new fishing gear. Meanwhile, in Nusa Penida, a MPA not in a UNESCO BR, 43% of the interviewees had increased income over the same period and none experienced a negative effect.

##### No change

Two studies, high validity and unclear validity reported no significant change. A survey by Bahadur et al. [2], rated as high validity, found that after 3 years of fishing reform (abolishment of fishing lots and the start of a community fishing policy) in Tonle Sap, for households dependent on fish, profit made from fishing, did not change significantly. Similarly, these households’ livelihood and incomes did not change significantly since the fishing reform. Authors suggest that complexities and trade-offs with alternative livelihoods may account for these non-significant findings.

Shively et al. [32] investigated the impacts of irrigating land in Palawan, comparing between irrigated and rain-fed sites, and before and after the irrigation. The study was rated unclear validity. Authors found a significant difference (at the p < 0.1 level) in income when comparing between irrigated and rain-fed lowland rice farms. More days of employment were available on upland farms after irrigation systems were added, compared to before, though this difference was not significant (p > 0.05).

##### Dependence on fishing

A survey by D’Agnes et al. [10], in Palawan, rated as high validity, found that coastal resource management (CRM) and reproductive health (RH) interventions had different impacts on the proportion of households being dependent on fishing. Specifically, there was no significant difference after CRM and RH were each implemented separately, however, when they were integrated as one package (CRM + RH), after at least 4 years, the proportion of households being dependent on fishing significantly decreased.

##### Livelihoods

The two studies (one moderate, one high validity) on livelihoods found no significant or positive impacts of interventions on livelihoods inside UNESCO BRs. The moderate validity study [15], compared the accessibility of alternative livelihoods between people living in Komodo National Park (UNESCO BR) and those in a community outside the BR but still in an MPA (Nusa Penida). In Komodo National Park, only 23% of the respondents had access to alternative livelihoods and their main obstacle was a lack of access to financial support to start businesses such as shops or street kitchens. In Nusa Penida, the access to additional sources of income was perceived as easy, especially tourism which was used by half of interviewees. Many interviewees said that their livelihoods depended on the region being protected, especially those working part-time in tourism. The survey in Torell et al. [36] (high validity) investigating the impacts of micro-credit intervention in Ranong BR (Thailand) found that there was no significant difference in the number of livelihoods per household between beneficiaries and non-beneficiaries (t = 1.328, df = 452, p > 0.1).

##### Employment

Indications are that there are positive employment outcomes across different intervention types, but due to the unclear and low validity of the two studies, conclusions cannot be drawn. The unclear validity study by Shively et al. [32], indicated that more days of employment were available on upland farms after irrigation systems were added, compared to before, though this difference was not significant. The low validity study by Nguyen [26] investigated the impacts of initiatives to aid local people in Cu Lao Cham island participating in tourism services and found that 90% of the respondents said that tourism provides jobs for the poor and 90% said it provides jobs for women. Nguyen [26] was rated as low validity because there were several external factors influencing the outcome, meaning that the training of people participating in tourism services was not the main factor in improving job opportunities.

##### Resource harvest and market

One moderate validity study indicated that whilst fish harvest was perceived to have decreased since MPAs began in the BR, the market for fish still exists. The study by Tupper et al. [38], found that in three sites of Palawan BR, local people gave an overall negative rating for "Perceptions of local resource harvest" between 2 and 4 years after MPA establishment. The fishers felt that target species were less available post-MPA establishment compared to before the MPAs were implemented. However, perceptions of the number and nature of markets were positive, indicating an awareness that there is still a market for the resource.

#### Material living standards

Five studies in our review reported on material living standards. One study was assigned high validity [2], two studies were assigned overall moderate validity [29, 39] one overall low validity [28] and one overall unclear [24]. Three studies used questionnaires/surveys, one using semi-structured questions, and two studies used interviews. The studies focused on food security, consumption and construction materials. In general, most interventions generated positive impacts on material living standards, with one high validity study showing a significant positive effect, though another showed no significant effect in the positive trend, both regarding food security, Fig. 5.

##### Food security

The survey by Ngoc [24], with unclear validity, reported that after 10 years’ implementation of MPA in Cu Lao Cham, most respondents somewhat agreed that the MPA had positive impacts on food security. The household survey at Tonle Sap Lake by Richardson [29], rated moderate validity, revealed that locals who continued on aquaculture programs had fewer problems feeding their families than those who discontinued (23% vs 40%) but the difference was not significant (z score = 1.381 p = 0.1673). The high validity study by Bahadur et al. [2] reported that after 3 years implementing community-based management in Tonle Sap, community members perceived there to be considerably better access to fish (p < 0.05), although livelihood and income did not change significantly (see above).

##### Consumption

Vong [39] conducted a study on household consumption of food, non-food (clothes, communication, and utility) and non-timber forest products in Tonle Sap Lake, comparing CBNRM households (Chiveng) and non-CBNRM households (Preak Sromoach). The study, rated moderate validity, found that among those who only fished within their community boundaries, those in CBNRM had a higher household consumption level than those in non-CBNRM areas, though significance levels were not tested.

##### Construction materials

The non-comparative study by Pido et al. [28] used household building materials as a proxy and found that after 6 years implementation of MPA in Palawan, the material style of life measures were: low 30.4%,middle 36.3%; and high 33.3%. However, this study was rated as low validity, because the parameter used (type of housing material) was only a proxy for material style of life, and due to external factors influencing the outcome.

#### Governance

We identified five studies in the review that considered governance as an outcome. Two studies were rated high validity [2, 36] two moderate validity [27, 38] and one unclear validity [24]. Four studies used questionnaires and one used workshops on perception. The five studies reported on different outcomes related to relations with protected area authorities, local participation and peoples’ perceptions on decision-making and legislation. For relations with local enforcement/government, results appear generally positive (though significance was not tested), across two high validity studies and one moderate validity study. An impact on participation in resource management was less well supported across two non-comparative studies (moderate and unclear validity).

Studies by Torell et al. [36] and Bahadur et al. [2], both high validity, revealed the improved relations between the stakeholders in the studied areas after the interventions. The non-comparative household survey by Torell et al. [36] reported that 51% of micro-credit beneficiaries agreed that the intervention improved their coordination with local governments. Similarly, the survey by Bahadur et al. [2] revealed that after 3 years’ implementation of CBNRM in Tonle Sap lake, 77.4% of respondents reported that conflict with law enforcement had significantly decreased.

The non-comparative study by Tupper et al. [38] investigated perception of MPA stakeholders in three sites of Palawan regarding the "Existence of a decision-making & management body", "Existence and adequacy of enabling legislation", and "Degree of interaction between managers & stakeholders". The study, rated moderate validity, found that across all three sites, these three aspects of governance were perceived as positive except for the last which was negative in one site, and positive in the other two sites.

The participation can occur in different ways. Palmer [27], describe a non-comparative study (survey), rated moderate validity, of community members in Lore Lindu national park (Indonesia) and found that after 14 years’ implementation, NGOs brought about different impacts on the participation of households in negotiating for co-management strategies. Under the influence of an NGO supporting indigenous rights to resource extraction, there was low participation of *all* household heads in co-management arrangements. Meanwhile, under the impacts of other NGOs focusing on biodiversity conservation, there were no co-management negotiations where *all* household heads participated, though the participation of *some* heads in the co-management negotiations was higher than for the areas where indigenous rights (Table 2). In contrast, in some cases, local participation can be limited as the survey (rated as unclear validity) by Ngoc [24] found that most fishing households were between "somewhat disagree" and "neither agree nor disagree" that they participated in creating/managing MPA regulation.

In general, the results mostly show that interventions can improve relationships between local people and local enforcement/government, Fig. 5.

#### Social relations

Six studies in our systematic review reported on social relations as outcomes of interventions. Two studies were assigned overall high validity [2, 36], three overall moderate validity [12, 15, 33] and one low validity [26]. Four studies used questionnaires and two used interviews. Most of the studies focus on social conflicts and social ties. One study explains the difference between gender in participating in community meetings.

The household survey by Torell et al. [36] found that a large percentage (73%) of micro-credit beneficiaries agreed that the intervention creates stronger social ties. The survey by Bahadur et al. [2] reported that after 3 years of fishing legislation in Tonle Sap, conflict between fishers significantly decreased (p < 0.1). Both these studies were rated as high validity. The study by Nguyen [26] revealed the percentage of tourism workers reporting conflicts in Cu Lao Cham: 29% said that tourism and initiatives by Cu Lao Cham Tourism Management board caused social conflicts between local tourism stakeholders and tour operators, 10% said it caused conflict between freelance guides and tour guides, and 12% said it caused conflict among local tourism stakeholders. Nguyen [26] was rated low validity due to several external factors influencing the outcome and a disconnect between the intervention’s impact on the outcome. The study by Dumlao [12], rated moderate validity, compared the tensions in local social relations between 2 groups: members and non-members of a mangrove rehabilitation project in Palawan, and found that in both groups, all respondents reported project-related social tensions (misunderstandings, boundary and area conflicts and lack of cooperation).

The fisherfolk survey by Sok et al. [33] in Tonle Sap Lake found that after 8 years of community fishing agreements, males were more likely to take part in community meetings and group discussions. Specifically, 46.3% of females compared to 75.2% of males participated in community meetings, 38.4% of females compared to 67.9% of males participated in group discussions, and 48.8% of females compared to 64.2% of males participated in workshops. The study was rated moderate validity and 301 people were surveyed.

The study by Eriksson et al. [15], rated moderate validity, investigated the impacts of resource use management project, by comparing a shark and manta ray conservation project in Komodo National Park and Nusa Penida (an MPA not within a UNESCO BR). The authors found that in Komodo, relations between fishing communities and conservation authorities & rangers were classed as poor by 67% of respondents. Only 20% of the respondents mentioned good relations. Some said that park rangers were threatening. Lack of dialogue for the zoning system led to past disputes. Meanwhile, in Nusa Penida, the majority (86%) of respondents had a good opinion of MPA management and boat patrols, seeing them as friendly, respectful, and trustworthy. They said that fishing had improved since protection from destructive fishing gear. We have categorised the outcomes described above as social relations because it focuses on the relationship between law enforcers and locals, though there are some aspects of described outcomes (e.g., opinion of MPA management) which may be viewed as relevant to governance outcomes.

Across the studies, which investigated a range of the interventions, it was shown that the marine management interventions can improve social ties, yet they may both reduce or cause social conflict, Fig. 5.

#### Health

One study reported on health outcomes, D’Agnes et al. [10], rated overall high validity. The survey compares youth contraceptive use at first sexual experience and young (15–24) males sexually active before and after the implementation of CRM and RH interventions. After at least 4 years’ implementation of separate CRM and RH interventions, there was no significant difference in either of the outcomes. However, when the two interventions were integrated, the contraceptive use of youth considerably increased and the proportion of young males sexually active significantly decreased, both trends in the desired direction.

#### Education

We identified two studies that reported education outcomes. One study was rated high validity, Torell et al. [36], and the other was moderate validity, Eriksson et al. [15]. The study designs limit the power and generalisability of the results, though it appears that interventions support development of skills and knowledge, whether in or out of a UNESCO BR.

The comparative study by Eriksson et al. [15] in Komodo National Park (UNESCO BR site) and Nusa Penida MPA (non-UNESCO BR site) found that in Komodo National Park, 43% of the 30 interviewees indicated a high degree of conservation-related knowledge and 40% a low degree. Many complained about poor access to information on regulations. This contrast with most (72%) of the 14 interviewees in Nusa Penida revealed high degrees of knowledge on the MPA and its regulations because stakeholder groups had been continually consulted and informed by the NGO managing the MPA implementation.

The non-comparative study by Torell et al. [36] in Ranong (Thailand) found that a large percentage (67%) of micro-credit beneficiaries agreed that the intervention helped develop their business skills.

There is no clear impact in either direction regarding education, though the one comparative study showed less knowledge in UNESCO BR compared to a non-BR. This lack of consistency in impact direction is perhaps predictable because each country or state has its own educational policies and that are not limited to, or wholly impacted by the BRs.

#### Subjective wellbeing

Of the two studies in our review which reported on subjective wellbeing, one study was assigned overall high validity [2] and one was low validity [26], and across both studies, the overall message is positive for subjective wellbeing of local people, Fig. 5.

The survey of 467 people by Bahadur et al. [2] in Tonle Sap reported that after 3 years of fishing legislation, people’s general happiness has improved and fear of getting into trouble has significantly decreased (p < 0.05).

Nguyen [26] interviewed local people working in the tourism sector in Cu Lao Cham (Vietnam) and found that 68% thought tourism improved perceived quality of life a lot, 15% thought it improved a little, and 17% thought there was no change. The rating of low validity was due to external factors influencing the outcome.

### Limitations of the evidence base

It was often difficult to determine whether studies fell within the zones or boundaries of UNESCO BRs, due to absence of publicly available maps of biosphere reserve areas, vague placements of study sites, and unclear descriptions of study locations. We used contacts within the UNESCO MAB network to determine wherever there was a lack of clarity.

Studies did not always describe in which zones the interventions were implemented. Accordingly, our review is not able to determine the impacts of interventions in different UNESCO BR zones (core, buffer and transition zones). Hence, in this review, we are unable to make comparisons between the impact of interventions in different zones, nor could the strength of regulations between different zones be compared. The following studies did not clarify which zone the intervention(s) were undertaken in: Bahadur [2, 12], Eriksson [15], Pido [28], Richardson [29], Shively [32], Sok [33], Torell [36, 39.

Many studies lacked detail about how data were gathered, raw data, or details about potential confounders and effect modifiers. We attempted to balance these reporting quality differences by contacting authors where possible to obtain further information. Where replies were received, information was helpful, though sometimes incomplete. The range of validity ratings demonstrated not only that better reporting is needed, but also an acknowledgement of the potentially large influence of confounding factors and effect modifiers on study outcomes. We are aware of some activities and interventions that were undertaken in BRs, which were otherwise eligible, but did not provide sufficient outcome information (e.g., separated socio-economic indicators) to be included in our review. Some of this ineligible evidence was reported in annual reports of the UNESCO BRs, which were searched as part of our comprehensive grey literature searching strategy.

### Limitations of this review

We searched only in the English language, due to language and resource constraints of the project and team and recognise the potential for material published in non-English languages to be missing from this review. Our searches were undertaken between November 2020 and January 2021, meaning that some of the searches will be more than 24 months old at the time of publication, including the journal publication processing time of more than 15 months. Due to the time and resource constraints of our funding, we are unable to update our searches, and recognise that new material may be available. Our full reporting of search strings, databases and grey literature sources enable others to repeat and update the evidence base.

Categorisation of the interventions was a challenge; we initially aimed to categorise the interventions according to the three UNESCO BR aims, but this was challenging due to several interventions targeting multiple aims, and that some interventions addressed a facet of one aim (the BR aims are multi-faceted, e.g., conservation of biodiversity and cultural diversity). Instead, we created categories that represented types of environmental management e.g., CBNRM, or human focus e.g., health, recognising that we would be unable to use these categories to map against the evidence base gaps.

The evidence base presented here is limited to describing interventions in UNESCO BRs and assessing the impact of the intervention itself. At times, the fact that an intervention was undertaken within a reserve may have had limited bearing on the success of the intervention, thus inferences about the effectiveness of UNESCO reserves cannot be drawn from any of the included studies.

Our validity assessment was designed to capture the potential for bias in the studies we identified, but as in any methodological assessment of quality was prone to subjectivity and may not have reflected the full differences in study quality across the heterogeneous studies that we encountered. This was compounded by the often lack of reporting on confounders and effect modifiers. We acknowledge these shortcomings: the validity summary was our attempt at transparency, yet we realise that wherever authors did not fully report on the potential for effect modifiers, our ratings may be impacted.

We did not assess whether the outcome we recorded was an intended outcome of the intervention. In several cases, these may have been explicitly stated in the paper, in others, it is highly likely, or assumed that the outcomes were intended, however in a few cases, the outcome may not have been intended, or may not have been the best measure of the interventions’ success. Post-hoc assessments of interventions undertaken by an external organisation are an example of this, and without further interrogation of published or, often, grey literature, the desired outcomes of the interventions can only be assumed.

Across all the outcomes we assessed, the wide range of different study designs, measures and outcome groups, along with the fact that many studies did not test the significance of the effects seen, make pooling the findings impossible and summarising impact challenging.

## Review conclusions

Across the studies presented here, we show that interventions implemented in UNESCO biosphere reserves can affect people in very different ways: having positive or negative impacts or seeming to affect no great change. Furthermore, in any one location, there may be a complex amalgamation of both positive and negative impacts, which may present across different socio-economic outcome domains. Indeed, conservation interventions can have a positive impact on the local economy, governance, social relations, subjective wellbeing, knowledge and health of local people. Economic living standards can be improved through job creation and alternative livelihoods [12, 37], reducing dependency on fishing [10] or better access to fish [2]. Some interventions were shown to contribute to improve the coordination of local people with local governance and strengthen social relations [2, 36], improve people’s general happiness [2], help local people have better knowledge related to conservation [15] and build business skills [36]. However, some of the studied interventions can also negatively impact other socio-economic aspects of the community, such as reducing access to resources for some members [15], increasing local social tensions related to boundaries and area conflicts, and failing to garner consensus on resource access and management plans [12, 15]. Moreover, some studies also revealed complex results between different stakeholders associated with an intervention. For example, Eriksson et al. [15] studied effects of conservation on economic income of people living in Komodo National Park (biosphere reserve) and Nusa Penida (outside biosphere reserve). In Komodo, 63% of the 30 respondents experienced reduced income because the zoning system complicated access to fishing grounds. Meanwhile, in Nusa Penida, 43% of 14 respondents had seen their income increase and no respondent reported a reduction in income. Many said that their livelihoods depend on the region being protected, especially those working part-time in tourism. Another example is the study of a Mangrove rehabilitation project in Palawan, Dumlao [12], in which 73% of members had uplift in family income through financial support by the project. In contrast, 23% of members said their income had decreased due to mangrove planting (e.g., time spent planting has low monetary return). This reconfirms that the expected win–win outcomes of UNESCO biosphere reserve model in terms of biodiversity and socio-economic development [3, 35] should be carefully considered, with particular respect to varied stakeholder groups, industries and temporal fluctuations in wellbeing. We emphasise the caveats presented in our limitations section, which recognise that the evidence presented here are a partial reflection of the real-world scenario.

CBNRM and livelihood interventions were most often studied, by eleven and ten articles, respectively. The studies showed that one CBNRM intervention can generate very different types of outcomes at the same time, specifically, improved governance, social relations and subjective wellbeing [2] and increased household consumption [39]. Especially, when being implemented together with a reproductive health intervention, CBNRM seemed to bring more positive impacts on local people’s health and decreased the household dependency on fishing [10].

Across the ten studies on livelihood, and focusing on those with moderate [29, 37] and high [36] validity, studies showed that livelihood interventions appear to generate positive impacts on material living standards [29], social relations, governance, education [36] and economic living standards [37].

The findings presented here are somewhat reflective of the existing evidence base e.g., that presented by a recent empirical study by Ruano-Chamorro et al. [30] on the varying socio-economic impact of the co-management of tropical coral reefs on fishers, the review by Gill et al. [17] on synergies, trade-offs and social impacts of marine conservation and the comprehensive review by Cox et al. [9] which synthesised studies evaluating Ostrom’s design principles for common-pool natural resource management. These and other works have, for some time, identified similar challenges and mechanisms for circumventing these challenges inherent in community-based natural resource management, which include, but are not limited to complexity, contextual importance, cultural nuances, collective choice and difficulties of monitoring. Thus, rather than breaking entirely novel ground, our systematic review serves as a reliable, transparently produced synthesis of the evidence in the UNESCO BR context that is in line with findings from more general reviews. More recent developments in the field have strongly challenged the win–win discourse on conservation and societal outcomes which has underlined many approaches to undertaking projects in the field [7, 22].

### Implications for policy/management

UNESCO BR is a designation to recognise and honour the biodiversity value of an area which is closely linked to local culture, livelihoods and/or wellbeing. Management mechanisms of the BRs are diverse and different from country to country, which is both a disadvantage and an advantage of this model. On the one hand, it can be very difficult to coordinate and connect stakeholders to implement activities in the biosphere reserve, promote the role and functions of a biosphere reserve. On the other hand, it is an open model that allows locals and nations build up a management mechanism flexibly to adapt to specific contexts.

Findings of this systematic review have pointed to several considerations with which policy makers may be able to improve the impact of BRs or of interventions within BRs and avoid some of the pitfalls which have led to negative or unchanged socio-economic outcomes for local populations. We suggest some points for consideration in the bullets below, particularly when developing management mechanisms for UNESCO biosphere reserves and/or managing activities in biosphere reserves. The involvement of the review team in UNESCO MAB in the SEA context means that these recommendations are directly available to stakeholders for discussion and potential implementation within the BR model. In particular, evidence clearly indicates that the livelihoods of local people must be considered before and during the implementation of an intervention, and where alternative livelihood is in planned, transitional support in terms of knowledge, finance, experience, etc. should be provided.

- Considering zoning policy to ensure people’s access to resources without causing negative impacts and difficulties [15].
- Focus on the relationship between management or government and the people to increase dialogue, respect and trust [12, 26, 15].
- Carefully considering the relationship of interests between stakeholders in a project to avoid causing social conflicts [12, 26, 15].
- Promoting dialogue with local people to equip them with more knowledge and understanding of conservation policy [15].
- Promoting the participation of people in the biosphere reserves to jointly building agreement, management and access to resources [26, 27].

We acknowledge that these recommendations reflect those already proposed in previous studies, reviews of evidence, and guidelines (Convention for Biological Diversity, IPBES) and are general rather than specific advice for policy makers and practitioners. Though we are unable to provide any ground-breaking or revolutionary suggestions, the implications for management presented here are grounded in the evidence base we present, and we do not attempt to extrapolate further than the findings allow.

### Implications for research

This systematic review has shown that the number of studies quantitatively examining the socio-economic impacts of the UNESCO biosphere reserves in Southeast Asia are very limited (16 studies to date). This confirms the urgent need for more studies on this topic to provide a thorough understanding of the relationship between nature conservation in general or BR model and socio-economic development. Qualitative data were excluded because the review focused on quantitative data only, without the resources to investigate qualitative data separately.

Several wellbeing outcomes were not found in our review (freedom of choice and action, security and safety, and culture and spirituality). This is likely because these outcomes are typically measured with qualitative methods while we only included quantitative and not qualitative data in this review. Further evidence synthesis with studies exploring these outcomes using quantitative approaches would be welcomed. A more standardised approach to measuring some outcomes, for example, validated and agreed measures, would aid comparability across different studies.

Establishing UNESCO BRs can take many years, and in such a timeframes, a variety of positive and negative impacts may arise, not least due to conflict between different interest groups. Interventions in biosphere reserves often address the trade-off between rapid socio-economic development based on resource extraction and long-term conservation of natural systems for long-term values. Many research studies are implemented in a short period (typically around 3 years, generally no more than 6 in this review), due to limited resources, and the types of studies featured in our review are no exception. Elucidating the long-term impacts of interventions undertaken in areas such as BRs with long term goals is highly unlikely in the timeframes of many research or monitoring projects, highlighting the need to initiate longer term monitoring projects appropriate to the timescales of such interventions.

Finally, as mentioned in the limitations of the evidence base, many studies lacked detail about research methodology (how data were gathered, raw data, potential confounders and effect modifiers). Therefore, we encourage authors of future studies to transparently report their methods and consider the influence of confounding factors and effect modifiers as well as including counterfactuals in research designs.

## Supplementary Information

**Additional file 1**: Database search methods details.

**Additional file 2**: Google and Google Scholar searches details.

**Additional file 3**: Theses and websites searches details.

**Additional file 4**: Records excluded at Full Text with reasons.

**Additional file 5**: Details of Consistency checking.

**Additional file 6**: Validity assessment.

**Additional file 7**: Data extraction spreadsheet.

**Additional file 8**: ROSES for Systematic Review Reports.

## Data Availability

All data generated or analysed during this study are included in this published article and its supplementary information files.

## Footnotes

1 http://www.unesco.org/new/en/natural-sciences/environment/ecological-sciences/biosphere-reserves/.

2 http://www.unesco.org/new/en/natural-sciences/environment/ecological-sciences/biosphere-reserves/asia-and-the-pacific/.

3 https://en.unesco.org/node/314143.

4 https://joannabriggs.org/sites/default/files/2019-05/JBI_Quasi-Experimental_Appraisal_Tool2017_0.pdf.

5 https://en.unesco.org/biosphere/designation#collapseSix.

6 https://en.unesco.org/sites/default/files/sc-21-conf-233-9_process_of_excellence_en.pdf.
