# Supplementary material for: What are the impacts of activities undertaken in UNESCO biosphere reserves on socio-economic wellbeing in Southeast Asia? A systematic review
Source: Environ Evid. 2023 Dec 14;12:30. doi: 10.1186/s13750-023-00322-1 (PMC11378852; doi:10.1186/s13750-023-00322-1)
Supplement: Supplementary file 1 — Additional file 1: Database search methods details. [file 13750_2023_322_MOESM1_ESM.docx]

**Additional File 1 Database Search Methods**

This file contains a list of test articles used to determine the sensitivity of searches, a list of multi-internationally designated sites within UNESCO biosphere reserves, and changes made to the search strategy, and the exact search strategies used in each of the databases.

Test articles

- D'Agnes, L., D’Agnes, H., Schwartz, J. B., Amarillo, M. L., & Castro, J. (2010). Integrated management of coastal resources and human health yields added value: a comparative study in Palawan (Philippines). Environmental Conservation, 398-409.
- Kuenzer, C., & Tuan, V. Q. (2013). Assessing the ecosystem services value of Can Gio Mangrove Biosphere Reserve: Combining earth-observation-and household-survey-based analyses. Applied Geography, 45, 167-184.
- Garces, L. R., Pido, M. D., Tupper, M. H., & Silvestre, G. T. (2013). Evaluating the management effectiveness of three marine protected areas in the Calamianes Islands, Palawan Province, Philippines: process, selected results and their implications for planning and management. Ocean & coastal management, 81, 49-57.
- Ngoc, Q. T. K. (2018). Impacts on the ecosystem and human well-being of the marine protected area in Cu Lao Cham, Vietnam. Marine Policy, 90, 174-183.
- Dygico, M., Songco, A., White, A. T., & Green, S. J. (2013). Achieving MPA effectiveness through application of responsive governance incentives in the Tubbataha reefs. Marine Policy, 41, 87-94.

List of multi-internationally designated sites within UNESCO biosphere reserves, and changes made to the search strategy.

- Boeng Chhmar and Associated River System and Floodplain RAMSAR site (within Tonle Sap, Cambodia)- we added “Boeng Chhmar” as a search term
- Prek Toal Ramsar Site RAMSAR site (within Tonle Sap, Cambodia) add “Prek Toal” as a search term
- Komodo National Park UNESCO World Heritage Site (within Komodo, Indonesia) – no additional search terms required
- Tropical Rainforest Heritage of Sumatra UNESCO World Heritage Site (within which Gunung Leuser is situated in Indonesia)- no additional search terms required
- Puerto Princesa Subterranean River National Park UNESCO World Heritage Site (within Palawan, Philippines)- we added “Puerto Princesa Subterranean River” as a search term
- Tubbataha Reefs National Park UNESCO World Heritage Site (within Palawan, Philippines)- we added “Tubbataha Reefs” as a search term
- Kaper Estuary - Laemson Marine National Park - Kraburi Estuary RAMSAR site (within Ranong, Thailand)- we added “Kaper Estuary” “Laemson Marine National Park” and “Kraburi Estuary” as search terms

**Medline (via Ovid)**

Searched on 8^th^ November 2020

("Tonle Sap" OR "Tonlé Sap" OR "Cibodas" OR "Komodo" OR "Lore Lindu" OR "Tanjung Puting" OR "Gunung Leuser" OR "Siberut" OR "Giam Siak Kecil-Bukit Batu" OR "Wakatobi" OR "Bromo Tengger Semeru*" OR "Taka Bonerate-Kepulauan Selayar" OR "Belambangan" OR "Berbak-Sembilang" OR "Betung Kerihun Danau Sentarum Kapuas Hulu" OR "Rinjani Lombok" OR "Tasik Chini" OR "Crocker Range" OR "Inlay Lake" OR "Inle Lake" OR "Indawgyi" OR "Puerto Galera" OR "Palawan" OR "Albay" OR "Sakaerat" OR "Hauy Tak Teak" OR "Haui Tak Teak" OR "Huai Tak Teak" OR "Mae Sa-Kog Ma" OR "Ranong" OR "Can Gio Mangrove" OR "Dong Nai" OR "Cat Ba" OR "Red River Delta" OR "Kien Giang" OR "Western Nghe An" OR "Mui Ca Mau" OR "Cu Lao Cham*" OR "Langbiang" OR "Boeng Chhmar" OR "Prek Toal" OR "Puerto Princesa Subterranean River" OR "Tubbataha Reefs" OR "Kaper Estuary" OR "Laemson Marine National Park" OR "Kraburi Estuary")

OR

"Biosphere reserve"

Use advanced search, keyword (which considers abstract, title and keywords) and no limits.

**Scopus**

Searched on 27^th^ January 2021

TITLE-ABS-KEY("Tonle Sap" OR "Tonlé Sap" OR "Cibodas" OR "Komodo" OR "Lore Lindu" OR "Tanjung Puting" OR "Gunung Leuser" OR "Siberut" OR "Giam Siak Kecil-Bukit Batu" OR "Wakatobi" OR "Bromo Tengger Semeru*" OR "Taka Bonerate-Kepulauan Selayar" OR "Belambangan" OR "Berbak-Sembilang" OR "Betung Kerihun Danau Sentarum Kapuas Hulu" OR "Rinjani Lombok" OR "Tasik Chini" OR "Crocker Range" OR "Inlay Lake" OR "Inle Lake" OR "Indawgyi" OR "Puerto Galera" OR "Palawan" OR "Albay" OR "Sakaerat" OR "Hauy Tak Teak" OR "Haui Tak Teak" OR "Huai Tak Teak" OR "Mae Sa-Kog Ma" OR "Ranong" OR "Can Gio Mangrove" OR "Dong Nai" OR "Cat Ba" OR "Red River Delta" OR "Kien Giang" OR "Western Nghe An" OR "Mui Ca Mau" OR "Cu Lao Cham*" OR "Langbiang" OR "Boeng Chhmar" OR "Prek Toal" OR "Puerto Princesa Subterranean River" OR "Tubbataha Reefs" OR "Kaper Estuary" OR "Laemson Marine National Park" OR "Kraburi Estuary") OR TITLE-ABS-KEY("biosphere reserve")

**Environment Complete.**

Searched on 12^th^ November 2020.

Advanced search, no limits

Because this database does not have a title abstract keywords option, we used two fields for each part of the search string:

**AB** ("Tonle Sap" OR "Tonlé Sap" OR "Cibodas" OR "Komodo" OR "Lore Lindu" OR "Tanjung Puting" OR "Gunung Leuser" OR "Siberut" OR "Giam Siak Kecil-Bukit Batu" OR "Wakatobi" OR "Bromo Tengger Semeru*" OR "Taka Bonerate-Kepulauan Selayar" OR "Belambangan" OR "Berbak-Sembilang" OR "Betung Kerihun Danau Sentarum Kapuas Hulu" OR "Rinjani Lombok" OR "Tasik Chini" OR "Crocker Range" OR "Inlay Lake" OR "Inle Lake" OR "Indawgyi" OR "Puerto Galera" OR "Palawan" OR "Albay" OR "Sakaerat" OR "Hauy Tak Teak" OR "Haui Tak Teak" OR "Huai Tak Teak" OR "Mae Sa-Kog Ma" OR "Ranong" OR "Can Gio Mangrove" OR "Dong Nai" OR "Cat Ba" OR "Red River Delta" OR "Kien Giang" OR "Western Nghe An" OR "Mui Ca Mau" OR "Cu Lao Cham*" OR "Langbiang" OR "Boeng Chhmar" OR "Prek Toal" OR "Puerto Princesa Subterranean River" OR "Tubbataha Reefs" OR "Kaper Estuary" OR "Laemson Marine National Park" OR "Kraburi Estuary")

OR

**AB** "Biosphere reserve"

OR

**TI** ("Tonle Sap" OR "Tonlé Sap" OR "Cibodas" OR "Komodo" OR "Lore Lindu" OR "Tanjung Puting" OR "Gunung Leuser" OR "Siberut" OR "Giam Siak Kecil-Bukit Batu" OR "Wakatobi" OR "Bromo Tengger Semeru*" OR "Taka Bonerate-Kepulauan Selayar" OR "Belambangan" OR "Berbak-Sembilang" OR "Betung Kerihun Danau Sentarum Kapuas Hulu" OR "Rinjani Lombok" OR "Tasik Chini" OR "Crocker Range" OR "Inlay Lake" OR "Inle Lake" OR "Indawgyi" OR "Puerto Galera" OR "Palawan" OR "Albay" OR "Sakaerat" OR "Hauy Tak Teak" OR "Haui Tak Teak" OR "Huai Tak Teak" OR "Mae Sa-Kog Ma" OR "Ranong" OR "Can Gio Mangrove" OR "Dong Nai" OR "Cat Ba" OR "Red River Delta" OR "Kien Giang" OR "Western Nghe An" OR "Mui Ca Mau" OR "Cu Lao Cham*" OR "Langbiang" OR "Boeng Chhmar" OR "Prek Toal" OR "Puerto Princesa Subterranean River" OR "Tubbataha Reefs" OR "Kaper Estuary" OR "Laemson Marine National Park" OR "Kraburi Estuary")

OR

**TI** "Biosphere reserve"

**Web of Science Core Collection**

Searched on 27^th^ January 2021

TS=("Tonle Sap" OR "Tonlé Sap" OR "Cibodas" OR "Komodo" OR "Lore Lindu" OR "Tanjung Puting" OR "Gunung Leuser" OR "Siberut" OR "Giam Siak Kecil$Bukit Batu" OR "Wakatobi" OR "Bromo Tengger Semeru*" OR "Taka Bonerate$Kepulauan Selayar" OR "Belambangan" OR "Berbak$Sembilang" OR "Betung Kerihun Danau Sentarum Kapuas Hulu" OR "Rinjani Lombok" OR "Tasik Chini" OR "Crocker Range" OR "Inlay Lake" OR "Inle Lake" OR "Indawgyi" OR "Puerto Galera" OR "Palawan" OR "Albay" OR "Sakaerat" OR "Hauy Tak Teak" OR "Haui Tak Teak" OR "Huai Tak Teak" OR "Mae Sa$Kog Ma" OR "Ranong" OR "Can Gio Mangrove" OR "Dong Nai" OR "Cat Ba" OR "Red River Delta" OR "Kien Giang" OR "Western Nghe An" OR "Mui Ca Mau" OR "Cu Lao Cham*" OR "Langbiang" OR "Boeng Chhmar" OR "Prek Toal" OR "Puerto Princesa Subterranean River" OR "Tubbataha Reefs" OR "Kaper Estuary" OR "Laemson Marine National Park" OR "Kraburi Estuary") OR TS=("Biosphere reserve")
