# Supplementary material for: What are the impacts of activities undertaken in UNESCO biosphere reserves on socio-economic wellbeing in Southeast Asia? A systematic review
Source: Environ Evid. 2023 Dec 14;12:30. doi: 10.1186/s13750-023-00322-1 (PMC11378852; doi:10.1186/s13750-023-00322-1)
Supplement: Supplementary file 2 — Additional file 2: Google and Google Scholar searches details. [file 13750_2023_322_MOESM2_ESM.docx]

**Additional file 2: Google Search (Advanced) Methods**

This file describes the methods for searching within Google for the systematic review.

Google advanced search on 14^th^ November 2020

Search string broken into 4 smaller strings because there is a limit of around 32 words in each Google search.

Use “with the extract phrase” and “with at least one of the words”

And limit to the “title of the page” to get more relevant results

This equates to a search that is essentially “biosphere reserve” AND “reserve names”

SEARCH 1

With the exact phrase:

“biosphere reserve”

With at least one of the words:

"Tonle Sap" "Tonlé Sap" "Cibodas" "Komodo" "Le Lindu" "Tanjung Puting" "Gunung Leuser" "Siberut" "Giam Siak Kecil-Bukit Batu" "Wakatobi" "Bromo Tengger Semeru*" "Taka Bonerate-Kepulauan Selayar"

SEARCH 2

With the exact phrase:

“biosphere reserve”

With at least one of the words:

"Belambangan" "Berbak-Sembilang" "Betung Kerihun Danau Sentarum Kapuas Hulu" "Rinjani Lombok" "Tasik Chini" "Crocker Range" "Inlay Lake" "Inle Lake" "Indawgyi" "Puerto Galera" "Palawan"

SEARCH 3

With the exact phrase:

“biosphere reserve”

With at least one of the words:

"Albay" "Sakaerat" "Hauy Tak Teak" "Haui Tak Teak" "Huai Tak Teak" "Mae Sa-Kog Ma" "Ranong" "Can Gio Mangrove" "Dong Nai" "Cat Ba" "Red River Delta" "Kien Giang" "Western Nghe An" "Mui Ca Mau"

SEARCH 4

With the exact phrase:

“biosphere reserve”

With at least one of the words:

"Cu Lao Cham*" "Langbiang" "Boeng Chhmar" "Prek Toal" "Puerto Princesa Subterranean River" "Tubbataha Reefs" "Kaper Estuary" "Laemson Marine National Park" "Kraburi Estuary"

Search results copied into a word file for screening.

**Google Scholar advanced search methods**

Followed the same as for the Google advanced search. Search results were downloaded as RIS files from Publish or Perish <https://harzing.com/resources/publish-or-perish>, to be later added to the database searches. Searches were undertaken on 15^th^ December 2020.
